# Chaotic vibration control of a composite cantilever beam

**DOI:** 10.1038/s41598-023-45113-3

**Published:** 2023-10-20

**Authors:** Xiaopei Liu, Lin Sun

**Affiliations:** School of Environmental and Safety Engineering, Liaoning Petrochemical University, Fushun, 113001 China

**Keywords:** Civil engineering, Mechanical engineering

## Abstract

In this research, an adaptive control strategy adapted from fuzzy sliding mode control is established and applied in chaotic vibration control of a multiple-dimension nonlinear dynamic system of a laminated composite cantilever beam. The third order shearing effect on the vibration of the beam is considered in the nonlinear dynamic model establishment, and the Hamilton principle as well as the Galerkin method is employed. It is discovered that a multi-dimensional nonlinear dynamic system of the cantilever beam needs to be considered for accurate vibration estimation. Therefore, the control strategy appropriate for the chaotic vibration control of a multiple-dimension system of the laminated composite beam is necessary, and then proves to be effective in chaotic vibration control in numerical simulation.

## Introduction

Advanced composite materials, featuring high strength, corrosion resistance, fatigue resistance and other advantages, are widely used in aerospace, civil engineering, mechanical engineering, and other engineering fields^1^. Some laminated composite structures, which can be simplified as cantilever beam models^2^, are applied in engineering components, such as aircraft wings, turbine engine blades, helicopter rotors and solar panels. However, the cantilever beams are prone to large deformation under external excitation, leading to nonlinear vibration problems that have negative impacts on the stability and safety of the system. Therefore, it is necessary to study the nonlinear vibration control of laminated composite cantilever beams.

Over the past decades, many researchers have studied the linear and nonlinear dynamics of cantilever beams. In general, linear structural models are constructed based on idealized engineering designs and they may not accurately represent all the aspects of the corresponding structures in practice^3^. Younis and Nayfeh^4^ proved that an inaccurate dynamic modeling of the system nonlinearities may result in an erroneous prediction of the dynamic behaviors. Particularly, nonlinear problems often occur in slender structures with large displacements, large rotations, and small strains. Bahari^3^ et al. have verified that the nonlinear analysis of a slender beam subjected to point load is closer to the experimental results comparing with the linear analysis.

Laplace transform and Adomian decomposition method (LADM) was employed to investigate semi-analytical solutions of Euler–Bernoulli beam equation in order to describe a uniform flexible cantilever beam^5^. Repka et al.^6^ applied the Timoshenko beam model in the analysis of the flexoelectric effect for a cantilever beam under large deformations, and considered the geometric nonlinearity with von Kármán strains. Meanwhile, some methods, such as a homotopy analysis method^7^, a rational elliptic balance method^8^, an enriched multiple scales method^9^, and an improved homotopy analysis method^10,11^, etc., have been gradually developed to solve nonlinear differential equations.

Naturally, researchers have also conducted a lot of research on nonlinear vibration of laminated composite cantilever beams. Roeser^12^ et al. developed the governing equations of motion of composite SPM/SPL cantilever beam based on the Euler–Bernoulli beam theory for transverse vibrations. Preethi et al.^13^ established the model of a nonuniform rotating laminated nano cantilever beam using the Timoshenko beam theory. Zhang et al.^2^ analyzed the nonlinear vibrations of laminated composite piezoelectric cantilever plates subjected to transverse and in-plane excitations based on Reddy’s third-order plate theory and Hamilton’s principle. Guo et al.^14^ considered Reddy’s third-order theory when conducting nonlinear dynamic analysis of macrofiber composite (MFC) laminated shells. Daros^15^ derived a fundamental solution for the harmonic vibration of asymmetrically laminated composite plates based on Reddy’s third-order shear deformation theory. Amabili et al.^16^ developed a refined third-order shear deformation theory to establish the model of a laminated composite beam and conducted an experimental verification. The studies above contribute to the nonlinear vibration control of cantilever structures considering 3rd order shearing effects.

In order to control vibrations of various nonlinear/linear dynamic systems derived from engineering^2,14,17–20^, different control strategies have been developed^21^. Among those control strategies, one strategy, namely the sliding mode control (SMC), was proposed in 1992 by Utkin^22^, and has been wildly applied in engineering vibration control along with other SMC based strategies. In 2019, Mobki et al.^23^ applied the SMC in a closed-loop control of a one-dimensional nonlinear dynamic system of a capacitive micro structure subjected to electrostatic forces; in 2020, Azizi^24^ used the SMC to reduce the unwanted vibrations of buildings subjected to earthquakes; in the next year, Azizi and Mobki^25^ employed the SMC for active control of car suspension systems. Based on the existing SMC, Mobki et al.^26^ designed an adaptive control scheme to control the vibration of a one-dimensional nonlinear dynamic system of a micro capacitor in 2020; in 2022, Azizi et al.^27^ also developed a nonsingular terminal SMC strategy to control the vibration of a one-dimensional nonlinear dynamic system of a micro structure. In order to mitigate the effects of uncertainties in dynamic systems, fuzzy rules were introduced into the traditional SMC in 2006, and hence a new control strategy namely the fuzzy sliding mode control (FSMC) was developed for Duffing-Holmes chaos synchronization with uncertainties^28^; in 2011, Yau et al.^29^ used the FSMC approach to control the chaotic vibration of a one-dimensional nonlinear dynamic system of a micro resonator; in 2022, Wu et al.^30^ applied the FSMC to stabilize Makovian jump nonlinear systems; in the same year, Ramakrishnan et al.^31^ also applied the FSMC to synchronize a chaotic oscillator in a fractional-order circuit. Based on the FSMC, Kuo^32^ proposed an adaptive FSMC for Sprott’s chaotic system synchronization in 2007; furthermore, Rajaei et al.^33^ developed an adaptive self-organizing FSMC scheme for a one-dimensional nonlinear dynamic system of a continuum nanobeam in 2022.

It should be noticed that: in the last decade, the existing FSMC based schemes can only be applied in nonlinear vibration control of one-dimensional dynamic systems of continuum structures, such as beams^29,33^. However, in the previous studies^2^, multi-dimensional nonlinear dynamic systems of continuum structures prove to be necessary in the investigations demanding accurate vibration estimation, especially in chaotic vibration investigations. Therefore, a control strategy is required, which can be applied in chaotic vibration control of multi-dimensional nonlinear dynamic systems of continuum structures. The control strategy to be presented in this research will contribute to the development of the FSMC based strategies by improving the previous application of the FSMC related schemes in nonlinear vibration control of continuum engineering structures^29,33^. Furthermore, the establishment of such control strategies may raise up a new research topic in nonlinear dynamics and control of continuum structures (i.e., strings, beams, plates, and shells). In this research, to control the nonlinear vibration of a multi-dimensional nonlinear dynamic system of a laminated composite continuum cantilever beam, a modified control strategy is proposed based on the FSMC. The governing equation of a laminated composite cantilever beam subjected to evenly distributed sinusoidal excitation is developed based on the Hamilton’s principle, and non-dimensional variables are then introduced into the governing equation. The Galerkin method is applied to derive a multi-dimensional nonlinear dynamic system of the cantilever beam. Then, based on the obtained multi-dimensional nonlinear dynamic system, numerical simulation is conducted to investigate the influence of higher vibration modes on the nonlinear dynamic behavior of the cantilever beam. Finally, the modified control strategy is established in response to the multi-dimensional nonlinear dynamic system, and then applied in controlling the chaotic vibration of the dynamic system to achieve vibration reduction in engineering fields.

## Model establishment

**Figure 1:**
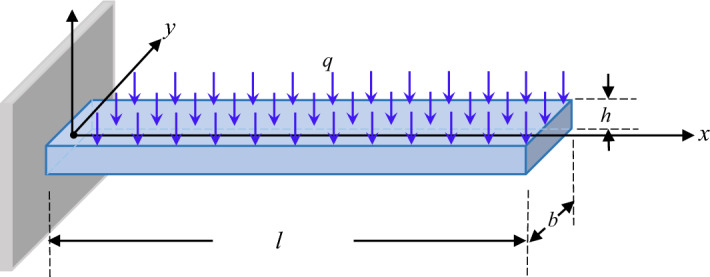
The sketch of the laminated composite cantilever beam.

In Fig. 1, the sketch of the 3-layer laminated composite cantilever beam is given. The beam features a uniform rectangular cross section; $l$, $b$, and $h$ represent its length, breadth, and thickness; $u_{0}$ and $w_{0}$ denote the displacements of any point in the middle plane of the cantilever beam along the *x*- and *z*- axes; a Cartesian coordinate is placed at the fixed end of the beam.

Before deformation, the position vector of a point $\left( {x,z} \right)$ of the beam is given as follows,

$$ {\mathbf{r}} = x{\mathbf{i}} + z{\mathbf{k}}, $$

where ${\mathbf{i}}$ and ${\mathbf{k}}$ represent the unit vectors of the Cartesian coordinate system.

Based on Reddy’s 3rd order shear deformation theory, the displacement of the beam is as follows,

$$ {\mathbf{R}} = \left( {x + u_{0} + z\phi_{x} - z^{3} c_{1} \left( {\phi_{x} + \frac{{\partial w_{0} }}{\partial x}} \right)} \right){{\mathbf i}} + \left( {z + w_{0} } \right){{\mathbf k}}, $$

where $c_{1} = {4 \mathord{\left/ {\vphantom {4 {\left( {3h^{2} } \right)}}} \right. \kern-0pt} {\left( {3h^{2} } \right)}}$, $u_{0}$ and $w_{0}$ represent the displacements along the *x*- and *z*- axes of any point on the mid-plane ($z = 0$), and $\phi_{x}$ is the slope of the deflection curve due to bending.

Therefore, the kinetic energy of the laminated composite beam is derived as,

1 $$ T = \int_{V} {\frac{1}{2}\rho } \left( {\frac{{d{\mathbf{R}}}}{dt}} \right)^{2} dV, $$

where $\rho$ is the density of the beam.

The von Kármán deformation associated with ${\mathbf{r}}$ is given as,

$$ \begin{aligned}&  \varepsilon_{11} = \frac{{\partial u_{0} }}{\partial x} + \frac{1}{2}\left( {\frac{{\partial w_{0} }}{\partial x}} \right)^{2} + z\frac{{\partial \phi_{x} }}{\partial x} - c_{1} z^{3} \left( {\frac{{\partial \phi_{x} }}{\partial x} + \frac{{\partial^{2} w_{0} }}{{\partial x^{2} }}} \right), \\ &\varepsilon_{13} = \left( {1 - 3c_{1} z^{2} } \right)\left( {\phi_{x} + \frac{{\partial w_{0} }}{\partial x}} \right).  \end{aligned} $$

Then, the strain energy of the beam can be obtained as,

2 $$ U = \int_{V} {\frac{1}{2}\left( {Q_{11} \varepsilon_{11} \varepsilon_{11} + Q_{13} \varepsilon_{13} \varepsilon_{13} } \right)dV} , $$

where $Q_{11}$ and $Q_{13}$ are the stiffness coefficients along the *x*- and *z*- axes.

The virtual work due to the external evenly distributed excitation $q$ and the damping effects is expressed as,

3 $$ W = b\int_{0}^{l} {qw_{0} \left( {x,t} \right)dx} - b\int_{0}^{l} {c\frac{{dw_{0} }}{dt}w_{0} dx} , $$

where $q = q_{0} \sin \omega t$, $q_{0}$ and $\omega$ represent the amplitude and the frequency of the sinusoidal excitation, and $c$ is the damping coefficient.

Following the Hamilton’s principle, it can be derived,

4 $$ \int_{{\;t_{1} }}^{{\;t_{2} }} {\;\;\left( {\delta L + \delta W} \right)\,} dt = 0, $$

where $$L = T - U$$.

Substitute Eq. (1), Eq. (2), and Eq. (3) into Eq. (4), it is derived as follows,

5 $$ \begin{aligned} & {\kern 1pt} {\kern 1pt} {\kern 1pt} {\kern 1pt} \int_{{{\kern 1pt} {\kern 1pt} {\kern 1pt} t{}_{1}}}^{{{\kern 1pt} {\kern 1pt} {\kern 1pt} t_{2} }} {{\kern 1pt} {\kern 1pt} {\kern 1pt} {\kern 1pt} \delta Ldt} = \int_{{{\kern 1pt} {\kern 1pt} {\kern 1pt} V}} {\int_{{{\kern 1pt} {\kern 1pt} {\kern 1pt} t_{1} }}^{{{\kern 1pt} {\kern 1pt} {\kern 1pt} t_{2} }} {{\kern 1pt} \left( {\rho \frac{{d{\mathbf{R}}}}{dt}} \right)\left( {\frac{{d\delta {\mathbf{R}}}}{dt}} \right)dt} dV} - \int_{{{\kern 1pt} {\kern 1pt} {\kern 1pt} t{}_{1}}}^{{{\kern 1pt} {\kern 1pt} {\kern 1pt} t_{2} }} {\int_{{{\kern 1pt} {\kern 1pt} {\kern 1pt} V}} {{\kern 1pt} {\kern 1pt} {\kern 1pt} \left( {{\kern 1pt} Q_{11} \varepsilon_{11} \delta \varepsilon_{11} + Q_{13} \varepsilon_{13} \delta \varepsilon_{13} } \right)dVdt} } \\ & = \int_{{{\kern 1pt} {\kern 1pt} {\kern 1pt} V}} {\left. {\left( {\rho \frac{{d{\mathbf{R}}}}{dt}\delta {\mathbf{R}}} \right)} \right|_{{{\mathbf{\mathop{R}\limits^{\rightharpoonup} }}_{{\mathbf{1}}} }}^{{{\mathbf{\mathop{R}\limits^{\rightharpoonup} }}_{{\mathbf{2}}} }} dV} - \int_{{{\kern 1pt} {\kern 1pt} {\kern 1pt} V}} {\int_{{{\kern 1pt} {\kern 1pt} {\kern 1pt} t_{1} }}^{{{\kern 1pt} {\kern 1pt} {\kern 1pt} t_{2} }} {{\kern 1pt} \rho \frac{{d^{2} {\mathbf{R}}}}{{dt^{2} }}\delta \mathop{R}\limits^{\rightharpoonup} dt} dV} \\ & \quad - \int_{{{\kern 1pt} {\kern 1pt} {\kern 1pt} t{}_{1}}}^{{{\kern 1pt} {\kern 1pt} {\kern 1pt} t_{2} }} {\int_{{{\kern 1pt} {\kern 1pt} {\kern 1pt} V}} {\left( {{\kern 1pt} Q_{11} \varepsilon_{11} \delta \varepsilon_{11} + Q_{13} \varepsilon_{13} \delta \varepsilon_{13} } \right)dVdt} }\\ & = 0 - \int_{{{\kern 1pt} {\kern 1pt} {\kern 1pt} V}} {\int_{{{\kern 1pt} {\kern 1pt} {\kern 1pt} t_{1} }}^{{{\kern 1pt} {\kern 1pt} {\kern 1pt} t_{2} }} {{\kern 1pt} \rho \frac{{d^{2} {\mathbf{R}}}}{{dt^{2} }}\delta \mathop{R}\limits^{\rightharpoonup} dt} dV} - \int_{{{\kern 1pt} {\kern 1pt} {\kern 1pt} t{}_{1}}}^{{{\kern 1pt} {\kern 1pt} {\kern 1pt} t_{2} }} {\int_{{{\kern 1pt} {\kern 1pt} {\kern 1pt} V}} {\left( {{\kern 1pt} Q_{11} \varepsilon_{11} \delta \varepsilon_{11} + Q_{13} \varepsilon_{13} \delta \varepsilon_{13} } \right)dVdt} } \\&= - b\int_{{{\kern 1pt} {\kern 1pt} {\kern 1pt} t_{1} }}^{{{\kern 1pt} {\kern 1pt} {\kern 1pt} t_{2} }} {\int_{{{\kern 1pt} {\kern 1pt} {\kern 1pt} - \frac{h}{2}}}^{{{\kern 1pt} {\kern 1pt} {\kern 1pt} \frac{h}{2}}} {\int_{{{\kern 1pt} {\kern 1pt} {\kern 1pt} 0}}^{{{\kern 1pt} {\kern 1pt} {\kern 1pt} l}} {{\kern 1pt} \rho \delta \left( {u\mathop{i}\limits^{\rightharpoonup} + w\mathop{k}\limits^{\rightharpoonup} } \right)\left( {\frac{{d^{2} u}}{{dt^{2} }}\mathop{i}\limits^{\rightharpoonup} + \frac{{d^{2} w}}{{dt^{2} }}\mathop{k}\limits^{\rightharpoonup} } \right)} } dxdzdt}\\ &\quad  - b\int_{{{\kern 1pt} {\kern 1pt} {\kern 1pt} t{}_{1}}}^{{{\kern 1pt} {\kern 1pt} {\kern 1pt} t_{2} }} {\int_{{{\kern 1pt} {\kern 1pt} {\kern 1pt} V}} {{\kern 1pt} Q_{11} } \varepsilon_{11} \delta \varepsilon_{11} dVdt} - b\int_{{{\kern 1pt} {\kern 1pt} {\kern 1pt} t{}_{1}}}^{{{\kern 1pt} {\kern 1pt} {\kern 1pt} t_{2} }} {\int_{{{\kern 1pt} {\kern 1pt} {\kern 1pt} V}} {{\kern 1pt} Q_{13} } \varepsilon_{13} \delta \varepsilon_{13} dVdt}\\ &  = - b\int_{{{\kern 1pt} {\kern 1pt} {\kern 1pt} t_{1} }}^{{{\kern 1pt} {\kern 1pt} {\kern 1pt} t_{2} }} {\int_{{{\kern 1pt} {\kern 1pt} {\kern 1pt} - \frac{h}{2}}}^{{{\kern 1pt} {\kern 1pt} {\kern 1pt} \frac{h}{2}}} {\int_{{{\kern 1pt} {\kern 1pt} {\kern 1pt} 0}}^{{{\kern 1pt} {\kern 1pt} {\kern 1pt} l}} {{\kern 1pt} \rho \delta \left( {x + u_{0} + z\phi_{x} - z^{3} c_{1} \left( {\phi_{x} + \frac{{\partial w_{0} }}{\partial x}} \right)} \right)\frac{{d^{2} u}}{{dt^{2} }}} } dxdzdt}\\ &  \quad - b\int_{{{\kern 1pt} {\kern 1pt} {\kern 1pt} t_{1} }}^{{{\kern 1pt} {\kern 1pt} {\kern 1pt} t_{2} }} {\int_{{{\kern 1pt} {\kern 1pt} {\kern 1pt} - \frac{h}{2}}}^{{{\kern 1pt} {\kern 1pt} {\kern 1pt} \frac{h}{2}}} {\int_{{{\kern 1pt} {\kern 1pt} {\kern 1pt} 0}}^{{{\kern 1pt} {\kern 1pt} {\kern 1pt} l}} {{\kern 1pt} \rho \delta \left( {z + w_{0} } \right)\frac{{d^{2} w_{0} }}{{dt^{2} }}} } dxdzdt}\\ & \quad - b\int_{{{\kern 1pt} {\kern 1pt} {\kern 1pt} t_{1} }}^{{{\kern 1pt} {\kern 1pt} {\kern 1pt} t_{2} }} {\int_{{{\kern 1pt} {\kern 1pt} {\kern 1pt} - \frac{h}{2}}}^{{{\kern 1pt} {\kern 1pt} {\kern 1pt} \frac{h}{2}}} {\int_{{{\kern 1pt} {\kern 1pt} {\kern 1pt} 0}}^{{{\kern 1pt} {\kern 1pt} {\kern 1pt} l}} {{\kern 1pt} Q_{11} \delta \left( {\frac{{\partial u_{0} }}{\partial x} + \frac{1}{2}\left( {\frac{{\partial w_{0} }}{\partial x}} \right)^{2} + z\frac{{\partial \phi_{x} }}{\partial x} - z^{3} c_{1} \left( {\frac{{\partial \phi_{x} }}{\partial x} + \frac{{\partial^{2} w_{0} }}{{\partial x^{2} }}} \right)} \right)\varepsilon_{11} } } dxdzdt}\\ &  \quad - b\int_{{{\kern 1pt} {\kern 1pt} {\kern 1pt} t_{1} }}^{{{\kern 1pt} {\kern 1pt} {\kern 1pt} t_{2} }} {\int_{{{\kern 1pt} {\kern 1pt} {\kern 1pt} - \frac{h}{2}}}^{{{\kern 1pt} {\kern 1pt} {\kern 1pt} \frac{h}{2}}} {\int_{{{\kern 1pt} {\kern 1pt} {\kern 1pt} 0}}^{{{\kern 1pt} {\kern 1pt} {\kern 1pt} l}} {{\kern 1pt} Q_{13} \delta \left( {1 - 3c_{1} z^{2} } \right)\left( {\frac{{\partial w_{0} }}{\partial x} + \phi_{x} } \right)\varepsilon_{13} } } dxdzdt} , \end{aligned} $$

where Eq. (5) can be presented as,

6a $$ L_{1}^{ * * } = - b\int_{{{\kern 1pt} {\kern 1pt} {\kern 1pt} t_{1} }}^{{{\kern 1pt} {\kern 1pt} {\kern 1pt} t_{2} }} {\int_{{{\kern 1pt} {\kern 1pt} {\kern 1pt} - \frac{h}{2}}}^{{{\kern 1pt} {\kern 1pt} {\kern 1pt} \frac{h}{2}}} {\int_{{{\kern 1pt} {\kern 1pt} {\kern 1pt} 0}}^{{{\kern 1pt} {\kern 1pt} {\kern 1pt} l}} {{\kern 1pt} \rho \delta \left( {x + u_{0} + z\phi_{x} - z^{3} c_{1} \left( {\phi_{x} + \frac{{\partial w_{0} }}{\partial x}} \right)} \right)\frac{{d^{2} u}}{{dt^{2} }}} } dxdzdt} , $$

6b $$ L_{2}^{ * * } = - b\int_{{{\kern 1pt} {\kern 1pt} {\kern 1pt} t_{1} }}^{{{\kern 1pt} {\kern 1pt} {\kern 1pt} t_{2} }} {\int_{{{\kern 1pt} {\kern 1pt} {\kern 1pt} - \frac{h}{2}}}^{{{\kern 1pt} {\kern 1pt} {\kern 1pt} \frac{h}{2}}} {\int_{{{\kern 1pt} {\kern 1pt} {\kern 1pt} 0}}^{{{\kern 1pt} {\kern 1pt} {\kern 1pt} l}} {{\kern 1pt} \rho \delta \left( {z + w_{0} } \right)\frac{{d^{2} w_{0} }}{{dt^{2} }}} } dxdzdt} , $$

6c $$ L_{3}^{ * * } = - b\int_{{{\kern 1pt} {\kern 1pt} {\kern 1pt} t_{1} }}^{{{\kern 1pt} {\kern 1pt} {\kern 1pt} t_{2} }} {\int_{{{\kern 1pt} {\kern 1pt} {\kern 1pt} - \frac{h}{2}}}^{{{\kern 1pt} {\kern 1pt} {\kern 1pt} \frac{h}{2}}} {\int_{{{\kern 1pt} {\kern 1pt} {\kern 1pt} 0}}^{{{\kern 1pt} {\kern 1pt} {\kern 1pt} l}} {{\kern 1pt} Q_{11} \delta \left( {\frac{{\partial u_{0} }}{\partial x} + \frac{1}{2}\left( {\frac{{\partial w_{0} }}{\partial x}} \right)^{2} + z\frac{{\partial \phi_{x} }}{\partial x} - z^{3} c_{1} \left( {\frac{{\partial \phi_{x} }}{\partial x} + \frac{{\partial^{2} w_{0} }}{{\partial x^{2} }}} \right)} \right)\varepsilon_{11} } } dxdzdt} , $$

6d $$ L_{4}^{ * * } = - b\int_{{{\kern 1pt} {\kern 1pt} {\kern 1pt} t_{1} }}^{{{\kern 1pt} {\kern 1pt} {\kern 1pt} t_{2} }} {\int_{{{\kern 1pt} {\kern 1pt} {\kern 1pt} - \frac{h}{2}}}^{{{\kern 1pt} {\kern 1pt} {\kern 1pt} \frac{h}{2}}} {\int_{{{\kern 1pt} {\kern 1pt} {\kern 1pt} 0}}^{{{\kern 1pt} {\kern 1pt} {\kern 1pt} l}} {{\kern 1pt} Q_{13} \delta \left( {1 - 3c_{1} z^{2} } \right)\left( {\frac{{\partial w_{0} }}{\partial x} + \phi_{x} } \right)\varepsilon_{13} } } dxdzdt} . $$

The equations in Eq. (6) can be further processed based on the features of the ortho-symmetric three-layer beam in the following,

7a $$ \begin{aligned}&  L_{1}^{ * * } = - b\int_{{{\kern 1pt} {\kern 1pt} {\kern 1pt} t_{1} }}^{{{\kern 1pt} {\kern 1pt} {\kern 1pt} t_{2} }} {\int_{{{\kern 1pt} {\kern 1pt} {\kern 1pt} - \frac{h}{2}}}^{{{\kern 1pt} {\kern 1pt} {\kern 1pt} \frac{h}{2}}} {\int_{{{\kern 1pt} {\kern 1pt} {\kern 1pt} 0}}^{{{\kern 1pt} {\kern 1pt} {\kern 1pt} l}} {{\kern 1pt} \rho \delta \left( {x + u_{0} + z\phi_{x} - z^{3} c_{1} \left( {\phi_{x} + \frac{{\partial w_{0} }}{\partial x}} \right)} \right)\frac{{d^{2} u}}{{dt^{2} }}} } dxdzdt}\\ &= - b\int_{{{\kern 1pt} {\kern 1pt} {\kern 1pt} t_{1} }}^{{{\kern 1pt} {\kern 1pt} {\kern 1pt} t_{2} }} {\int_{{{\kern 1pt} {\kern 1pt} {\kern 1pt} - \frac{h}{2}}}^{{{\kern 1pt} {\kern 1pt} {\kern 1pt} \frac{h}{2}}} {\int_{{{\kern 1pt} {\kern 1pt} {\kern 1pt} 0}}^{{{\kern 1pt} {\kern 1pt} {\kern 1pt} l}} {{\kern 1pt} {\kern 1pt} \rho \frac{{d^{2} u}}{{dt^{2} }}\delta u_{0} } } dxdzdt} {\kern 1pt} - b\int_{{{\kern 1pt} {\kern 1pt} {\kern 1pt} t_{1} }}^{{{\kern 1pt} {\kern 1pt} {\kern 1pt} t_{2} }} {\int_{{{\kern 1pt} {\kern 1pt} {\kern 1pt} - \frac{h}{2}}}^{{{\kern 1pt} {\kern 1pt} {\kern 1pt} \frac{h}{2}}} {\int_{{{\kern 1pt} {\kern 1pt} {\kern 1pt} 0}}^{{{\kern 1pt} {\kern 1pt} {\kern 1pt} l}} {{\kern 1pt} {\kern 1pt} \rho z\frac{{d^{2} u}}{{dt^{2} }}\delta \phi_{x} } } dxdzdt}\\ &   \quad - b\int_{{{\kern 1pt} {\kern 1pt} {\kern 1pt} t_{1} }}^{{{\kern 1pt} {\kern 1pt} {\kern 1pt} t_{2} }} {\int_{{{\kern 1pt} {\kern 1pt} {\kern 1pt} - \frac{h}{2}}}^{{{\kern 1pt} {\kern 1pt} {\kern 1pt} \frac{h}{2}}} {\int_{{{\kern 1pt} {\kern 1pt} {\kern 1pt} 0}}^{{{\kern 1pt} {\kern 1pt} {\kern 1pt} l}} {{\kern 1pt} \left( { - \rho z^{3} c_{1} \left( {\frac{{d^{2} u}}{{dt^{2} }}\delta \phi_{x} } \right)} \right)} } dxdzdt}\\ & \quad - b\int_{{{\kern 1pt} {\kern 1pt} {\kern 1pt} t_{1} }}^{{{\kern 1pt} {\kern 1pt} {\kern 1pt} t_{2} }} {\int_{{{\kern 1pt} {\kern 1pt} {\kern 1pt} - \frac{h}{2}}}^{{{\kern 1pt} {\kern 1pt} {\kern 1pt} \frac{h}{2}}} {\int_{{{\kern 1pt} {\kern 1pt} {\kern 1pt} 0}}^{{{\kern 1pt} {\kern 1pt} {\kern 1pt} l}} {{\kern 1pt} \left( { - \rho z^{3} c_{1} \left( {\frac{{d^{2} u}}{{dt^{2} }}\frac{{\partial \delta w_{0} }}{\partial x}} \right)} \right)} } dxdzdt}\\ &= - b\int_{{{\kern 1pt} {\kern 1pt} {\kern 1pt} t_{1} }}^{{{\kern 1pt} {\kern 1pt} {\kern 1pt} t_{2} }} {\int_{{{\kern 1pt} {\kern 1pt} {\kern 1pt} - \frac{h}{2}}}^{{{\kern 1pt} {\kern 1pt} {\kern 1pt} \frac{h}{2}}} {\int_{{{\kern 1pt} {\kern 1pt} {\kern 1pt} 0}}^{{{\kern 1pt} {\kern 1pt} {\kern 1pt} l}} {{\kern 1pt} {\kern 1pt} \rho \frac{{d^{2} u}}{{dt^{2} }}\delta u_{0} } } dxdzdt} - b\int_{{{\kern 1pt} {\kern 1pt} {\kern 1pt} t_{1} }}^{{{\kern 1pt} {\kern 1pt} {\kern 1pt} t_{2} }} {\int_{{{\kern 1pt} {\kern 1pt} {\kern 1pt} - \frac{h}{2}}}^{{{\kern 1pt} {\kern 1pt} {\kern 1pt} \frac{h}{2}}} {\int_{{{\kern 1pt} {\kern 1pt} {\kern 1pt} 0}}^{{{\kern 1pt} {\kern 1pt} {\kern 1pt} l}} {{\kern 1pt} {\kern 1pt} \rho z\frac{{d^{2} u}}{{dt^{2} }}\delta \phi_{x} } } dxdzdt} \\ & \quad  - b\int_{{{\kern 1pt} {\kern 1pt} {\kern 1pt} t_{1} }}^{{{\kern 1pt} {\kern 1pt} {\kern 1pt} t_{2} }} {\int_{{{\kern 1pt} {\kern 1pt} {\kern 1pt} - \frac{h}{2}}}^{{{\kern 1pt} {\kern 1pt} {\kern 1pt} \frac{h}{2}}} {\int_{{{\kern 1pt} {\kern 1pt} {\kern 1pt} 0}}^{{{\kern 1pt} {\kern 1pt} {\kern 1pt} l}} {{\kern 1pt} \left( { - \rho z^{3} c_{1} \left( {\frac{{d^{2} u}}{{dt^{2} }}\delta \phi_{x} } \right)} \right)} } dxdzdt}\\ & 0 - b\int_{{{\kern 1pt} {\kern 1pt} {\kern 1pt} t_{1} }}^{{{\kern 1pt} {\kern 1pt} {\kern 1pt} t_{2} }} {\int_{{{\kern 1pt} {\kern 1pt} {\kern 1pt} - \frac{h}{2}}}^{{{\kern 1pt} {\kern 1pt} {\kern 1pt} \frac{h}{2}}} {\int_{{{\kern 1pt} {\kern 1pt} {\kern 1pt} 0}}^{{{\kern 1pt} {\kern 1pt} {\kern 1pt} l}} {{\kern 1pt} \left( {\rho z^{3} c_{1} \frac{\partial }{\partial x}\left( {\frac{{d^{2} u}}{{dt^{2} }}} \right)} \right)} } \delta w_{0} dxdzdt}\\ &= - b\int_{{{\kern 1pt} {\kern 1pt} {\kern 1pt} t_{1} }}^{{{\kern 1pt} {\kern 1pt} {\kern 1pt} t_{2} }} {\int_{{{\kern 1pt} {\kern 1pt} {\kern 1pt} - \frac{h}{2}}}^{{{\kern 1pt} {\kern 1pt} {\kern 1pt} \frac{h}{2}}} {\int_{{{\kern 1pt} {\kern 1pt} {\kern 1pt} 0}}^{{{\kern 1pt} {\kern 1pt} {\kern 1pt} l}} {{\kern 1pt} {\kern 1pt} \rho \frac{{d^{2} }}{{dt^{2} }}\left( {x + u_{0} + z\phi_{x} - z^{3} c_{1} \left( {\phi_{x} + \frac{{\partial w_{0} }}{\partial x}} \right)} \right)\delta u_{0} } } dxdzdt}  \\ & \quad- b\int_{{{\kern 1pt} {\kern 1pt} {\kern 1pt} t_{1} }}^{{{\kern 1pt} {\kern 1pt} {\kern 1pt} t_{2} }} {\int_{{{\kern 1pt} {\kern 1pt} {\kern 1pt} - \frac{h}{2}}}^{{{\kern 1pt} {\kern 1pt} {\kern 1pt} \frac{h}{2}}} {\int_{{{\kern 1pt} {\kern 1pt} {\kern 1pt} 0}}^{{{\kern 1pt} {\kern 1pt} {\kern 1pt} l}} {{\kern 1pt} {\kern 1pt} \rho z\frac{{d^{2} }}{{dt^{2} }}\left( {x + u_{0} + z\phi_{x} - z^{3} c_{1} \left( {\phi_{x} + \frac{{\partial w_{0} }}{\partial x}} \right)} \right)\delta \phi_{x} } } dxdzdt}\\ &\quad - b\int_{{{\kern 1pt} {\kern 1pt} {\kern 1pt} t_{1} }}^{{{\kern 1pt} {\kern 1pt} {\kern 1pt} t_{2} }} {\int_{{{\kern 1pt} {\kern 1pt} {\kern 1pt} - \frac{h}{2}}}^{{{\kern 1pt} {\kern 1pt} {\kern 1pt} \frac{h}{2}}} {\int_{{{\kern 1pt} {\kern 1pt} {\kern 1pt} 0}}^{{{\kern 1pt} {\kern 1pt} {\kern 1pt} l}} {{\kern 1pt} \left( { - \rho z^{3} c_{1} \left( {\frac{{d^{2} }}{{dt^{2} }}\left( {x + u_{0} + z\phi_{x} - z^{3} c_{1} \left( {\phi_{x} + \frac{{\partial w_{0} }}{\partial x}} \right)} \right)\delta \phi_{x} } \right)} \right)} } dxdzdt}\\ & \quad   - b\int_{{{\kern 1pt} {\kern 1pt} {\kern 1pt} t_{1} }}^{{{\kern 1pt} {\kern 1pt} {\kern 1pt} t_{2} }} {\int_{{{\kern 1pt} {\kern 1pt} {\kern 1pt} - \frac{h}{2}}}^{{{\kern 1pt} {\kern 1pt} {\kern 1pt} \frac{h}{2}}} {\int_{{{\kern 1pt} {\kern 1pt} {\kern 1pt} 0}}^{{{\kern 1pt} {\kern 1pt} {\kern 1pt} l}} {{\kern 1pt} \rho z^{3} c_{1} \frac{\partial }{\partial x}\left( {\frac{{d^{2} }}{{dt^{2} }}\left( {x + u_{0} + z\phi_{x} - z^{3} c_{1} \left( {\phi_{x} + \frac{{\partial w_{0} }}{\partial x}} \right)} \right)} \right)} } \delta w_{0} dxdzdt}\\ & = - b\int_{{{\kern 1pt} {\kern 1pt} {\kern 1pt} t_{1} }}^{{{\kern 1pt} {\kern 1pt} {\kern 1pt} t_{2} }} {\int_{{{\kern 1pt} {\kern 1pt} {\kern 1pt} - \frac{h}{2}}}^{{{\kern 1pt} {\kern 1pt} {\kern 1pt} \frac{h}{2}}} {\int_{{{\kern 1pt} {\kern 1pt} {\kern 1pt} 0}}^{{{\kern 1pt} {\kern 1pt} {\kern 1pt} l}} {{\kern 1pt} {\kern 1pt} \rho \frac{{d^{2} }}{{dt^{2} }}\left( {x + u_{0} } \right)\delta u_{0} } } dxdzdt} \\ & \quad - b\int_{{{\kern 1pt} {\kern 1pt} {\kern 1pt} t_{1} }}^{{{\kern 1pt} {\kern 1pt} {\kern 1pt} t_{2} }} {\int_{{{\kern 1pt} {\kern 1pt} {\kern 1pt} - \frac{h}{2}}}^{{{\kern 1pt} {\kern 1pt} {\kern 1pt} \frac{h}{2}}} {\int_{{{\kern 1pt} {\kern 1pt} {\kern 1pt} 0}}^{{{\kern 1pt} {\kern 1pt} {\kern 1pt} l}} {{\kern 1pt} {\kern 1pt} \rho z\frac{{d^{2} }}{{dt^{2} }}\left( {z\phi_{x} - z^{3} c_{1} \left( {\phi_{x} + \frac{{\partial w_{0} }}{\partial x}} \right)} \right)\delta \phi_{x} } } dxdzdt}\\ & \quad    - b\int_{{{\kern 1pt} {\kern 1pt} {\kern 1pt} t_{1} }}^{{{\kern 1pt} {\kern 1pt} {\kern 1pt} t_{2} }} {\int_{{{\kern 1pt} {\kern 1pt} {\kern 1pt} - \frac{h}{2}}}^{{{\kern 1pt} {\kern 1pt} {\kern 1pt} \frac{h}{2}}} {\int_{{{\kern 1pt} {\kern 1pt} {\kern 1pt} 0}}^{{{\kern 1pt} {\kern 1pt} {\kern 1pt} l}} {{\kern 1pt} \left( { - \rho z^{3} c_{1} \left( {\frac{{d^{2} }}{{dt^{2} }}\left( {z\phi_{x} - z^{3} c_{1} \left( {\phi_{x} + \frac{{\partial w_{0} }}{\partial x}} \right)} \right)\delta \phi_{x} } \right)} \right)} } dxdzdt}\\ & \quad - b\int_{{{\kern 1pt} {\kern 1pt} {\kern 1pt} t_{1} }}^{{{\kern 1pt} {\kern 1pt} {\kern 1pt} t_{2} }} {\int_{{{\kern 1pt} {\kern 1pt} {\kern 1pt} - \frac{h}{2}}}^{{{\kern 1pt} {\kern 1pt} {\kern 1pt} \frac{h}{2}}} {\int_{{{\kern 1pt} {\kern 1pt} {\kern 1pt} 0}}^{{{\kern 1pt} {\kern 1pt} {\kern 1pt} l}} {{\kern 1pt} \rho z^{3} c_{1} \frac{\partial }{\partial x}\left( {\frac{{d^{2} }}{{dt^{2} }}\left( {z\phi_{x} - z^{3} c_{1} \left( {\phi_{x} + \frac{{\partial w_{0} }}{\partial x}} \right)} \right)} \right)} } \delta w_{0} dxdzdt} , \end{aligned}$$

7b $$\begin{aligned}& L_{2}^{ * * } = - b\int_{{{\kern 1pt} {\kern 1pt} {\kern 1pt} t_{1} }}^{{{\kern 1pt} {\kern 1pt} {\kern 1pt} t_{2} }} {\int_{{{\kern 1pt} {\kern 1pt} {\kern 1pt} - \frac{h}{2}}}^{{{\kern 1pt} {\kern 1pt} {\kern 1pt} \frac{h}{2}}} {\int_{{{\kern 1pt} {\kern 1pt} {\kern 1pt} 0}}^{{{\kern 1pt} {\kern 1pt} {\kern 1pt} l}} {{\kern 1pt} \rho \delta \left( {z + w_{0} } \right)\frac{{d^{2} w_{0} }}{{dt^{2} }}} } dxdzdt}\\ &= - b\int_{{{\kern 1pt} {\kern 1pt} {\kern 1pt} t_{1} }}^{{{\kern 1pt} {\kern 1pt} {\kern 1pt} t_{2} }} {\int_{{{\kern 1pt} {\kern 1pt} {\kern 1pt} - \frac{h}{2}}}^{{{\kern 1pt} {\kern 1pt} \frac{h}{2}}} {\int_{{{\kern 1pt} {\kern 1pt} {\kern 1pt} 0}}^{{{\kern 1pt} {\kern 1pt} {\kern 1pt} l}} {{\kern 1pt} \rho \frac{{d^{2} w_{0} }}{{dt^{2} }}\delta w_{0} } } dxdzdt} ,  \end{aligned} $$

7c $$ \begin{aligned} & L_{3}^{ * * } = - b\int_{{{\kern 1pt} {\kern 1pt} {\kern 1pt} t_{1} }}^{{{\kern 1pt} {\kern 1pt} {\kern 1pt} t_{2} }} {\int_{{{\kern 1pt} {\kern 1pt} {\kern 1pt} - \frac{h}{2}}}^{{{\kern 1pt} {\kern 1pt} {\kern 1pt} \frac{h}{2}}} {\int_{{{\kern 1pt} {\kern 1pt} {\kern 1pt} 0}}^{{{\kern 1pt} {\kern 1pt} {\kern 1pt} l}} {{\kern 1pt} Q_{11} \delta \left( {\frac{{\partial u_{0} }}{\partial x} + \frac{1}{2}\left( {\frac{{\partial w_{0} }}{\partial x}} \right)^{2} + z\frac{{\partial \phi_{x} }}{\partial x} - z^{3} c_{1} \left( {\frac{{\partial \phi_{x} }}{\partial x} + \frac{{\partial^{2} w_{0} }}{{\partial x^{2} }}} \right)} \right)\varepsilon_{11} } } dxdzdt}\\ &  = - \left( {0 - b\int_{{{\kern 1pt} {\kern 1pt} {\kern 1pt} t_{1} }}^{{{\kern 1pt} {\kern 1pt} {\kern 1pt} t_{2} }} {\int_{{{\kern 1pt} {\kern 1pt} {\kern 1pt} - \frac{h}{2}}}^{{{\kern 1pt} {\kern 1pt} {\kern 1pt} \frac{h}{2}}} {\int_{{{\kern 1pt} {\kern 1pt} {\kern 1pt} 0}}^{{{\kern 1pt} {\kern 1pt} {\kern 1pt} l}} {{\kern 1pt} Q_{11} \frac{{\partial \varepsilon_{11} }}{\partial x}\delta u_{0} dx} } dzdt} } \right) \\ & \quad - \left( {0 - b\int_{{{\kern 1pt} {\kern 1pt} {\kern 1pt} t_{1} }}^{{{\kern 1pt} {\kern 1pt} {\kern 1pt} t_{2} }} {\int_{{{\kern 1pt} {\kern 1pt} {\kern 1pt} - \frac{h}{2}}}^{{{\kern 1pt} {\kern 1pt} {\kern 1pt} \frac{h}{2}}} {\int_{{{\kern 1pt} {\kern 1pt} {\kern 1pt} 0}}^{{{\kern 1pt} {\kern 1pt} {\kern 1pt} l}} {{\kern 1pt} Q_{11} \frac{\partial }{\partial x}\left( {\varepsilon_{11} \frac{{\partial w_{0} }}{\partial x}} \right)\delta w_{0} dx} } dzdt} } \right) \\ & \quad - \left( {0 - b\int_{{{\kern 1pt} {\kern 1pt} {\kern 1pt} t_{1} }}^{{{\kern 1pt} {\kern 1pt} {\kern 1pt} t_{2} }} {\int_{{{\kern 1pt} {\kern 1pt} {\kern 1pt} - \frac{h}{2}}}^{{{\kern 1pt} {\kern 1pt} {\kern 1pt} \frac{h}{2}}} {\int_{{{\kern 1pt} {\kern 1pt} {\kern 1pt} 0}}^{{{\kern 1pt} {\kern 1pt} {\kern 1pt} l}} {{\kern 1pt} zQ_{11} \frac{{\partial \varepsilon_{11} }}{\partial x}\delta \phi_{x} } } dxdzdt} } \right)\\ &  \quad - \left( {0 - b\int_{{{\kern 1pt} {\kern 1pt} {\kern 1pt} t_{1} }}^{{{\kern 1pt} {\kern 1pt} {\kern 1pt} t_{2} }} {\int_{{{\kern 1pt} {\kern 1pt} {\kern 1pt} - \frac{h}{2}}}^{{{\kern 1pt} {\kern 1pt} {\kern 1pt} \frac{h}{2}}} {\int_{{{\kern 1pt} {\kern 1pt} {\kern 1pt} 0}}^{{{\kern 1pt} {\kern 1pt} {\kern 1pt} l}} {{\kern 1pt} \left( { - z^{3} c_{1} Q_{11} \frac{{\partial \varepsilon_{11} }}{\partial x}} \right)} } \delta \phi_{x} dxdzdt} } \right) \\ & \quad  {\kern 1pt} {\kern 1pt} {\kern 1pt} - \left( {0 - \left( {0 - b\int_{{{\kern 1pt} {\kern 1pt} {\kern 1pt} t_{1} }}^{{{\kern 1pt} {\kern 1pt} {\kern 1pt} t_{2} }} {\int_{{{\kern 1pt} {\kern 1pt} {\kern 1pt} - \frac{h}{2}}}^{{{\kern 1pt} {\kern 1pt} {\kern 1pt} \frac{h}{2}}} {\int_{{{\kern 1pt} 0}}^{{{\kern 1pt} l}} {{\kern 1pt} \left( { - z^{3} c_{1} Q_{11} \frac{{\partial^{2} \varepsilon_{11} }}{{\partial x^{2} }}} \right)} } \delta w_{0} dxdzdt} } \right)} \right) \\ &= b\int_{{{\kern 1pt} {\kern 1pt} {\kern 1pt} t_{1} }}^{{{\kern 1pt} {\kern 1pt} {\kern 1pt} t_{2} }} {\int_{{{\kern 1pt} {\kern 1pt} {\kern 1pt} - \frac{h}{2}}}^{{{\kern 1pt} {\kern 1pt} {\kern 1pt} \frac{h}{2}}} {\int_{{{\kern 1pt} {\kern 1pt} {\kern 1pt} 0}}^{{{\kern 1pt} {\kern 1pt} {\kern 1pt} l}} {{\kern 1pt} Q_{11} \frac{\partial }{\partial x}\left( {\frac{{\partial u_{0} }}{\partial x} + \frac{1}{2}\left( {\frac{{\partial w_{0} }}{\partial x}} \right)^{2} + z\frac{{\partial \phi_{x} }}{\partial x} - z^{3} c_{1} \left( {\frac{{\partial \phi_{x} }}{\partial x} + \frac{{\partial^{2} w_{0} }}{{\partial x^{2} }}} \right)} \right)\delta u_{0} dx} } dzdt}\\  & \quad + b\int_{{{\kern 1pt} {\kern 1pt} {\kern 1pt} t_{1} }}^{{{\kern 1pt} {\kern 1pt} {\kern 1pt} {\kern 1pt} t_{2} }} {\int_{{{\kern 1pt} {\kern 1pt} {\kern 1pt} - \frac{h}{2}}}^{{{\kern 1pt} {\kern 1pt} {\kern 1pt} \frac{h}{2}}} {\int_{{{\kern 1pt} {\kern 1pt} {\kern 1pt} 0}}^{{{\kern 1pt} {\kern 1pt} {\kern 1pt} l}} {{\kern 1pt} Q_{11} \frac{\partial }{\partial x}\left( {\frac{{\partial u_{0} }}{\partial x} + \frac{1}{2}\left( {\frac{{\partial w_{0} }}{\partial x}} \right)^{2} + z\frac{{\partial \phi_{x} }}{\partial x} - z^{3} c_{1} \left( {\frac{{\partial \phi_{x} }}{\partial x} + \frac{{\partial^{2} w_{0} }}{{\partial x^{2} }}} \right)} \right)\frac{{\partial w_{0} }}{\partial x}\delta w_{0} } } dxdzdt} \\ &\quad    + b\int_{{{\kern 1pt} {\kern 1pt} {\kern 1pt} t_{1} }}^{{{\kern 1pt} {\kern 1pt} {\kern 1pt} t_{2} }} {\int_{{{\kern 1pt} {\kern 1pt} {\kern 1pt} - \frac{h}{2}}}^{{{\kern 1pt} {\kern 1pt} {\kern 1pt} \frac{h}{2}}} {\int_{{{\kern 1pt} {\kern 1pt} {\kern 1pt} 0}}^{{{\kern 1pt} {\kern 1pt} {\kern 1pt} l}} {{\kern 1pt} Q_{11} \left( {\frac{{\partial u_{0} }}{\partial x} + \frac{1}{2}\left( {\frac{{\partial w_{0} }}{\partial x}} \right)^{2} + z\frac{{\partial \phi_{x} }}{\partial x} - z^{3} c_{1} \left( {\frac{{\partial \phi_{x} }}{\partial x} + \frac{{\partial^{2} w_{0} }}{{\partial x^{2} }}} \right)} \right)\frac{{\partial^{2} w_{0} }}{{\partial x^{2} }}} } \delta w_{0} dxdzdt} \\ & \quad  + b\int_{{{\kern 1pt} {\kern 1pt} {\kern 1pt} t_{1} }}^{{{\kern 1pt} {\kern 1pt} {\kern 1pt} t_{2} }} {\int_{{{\kern 1pt} {\kern 1pt} {\kern 1pt} - \frac{h}{2}}}^{{{\kern 1pt} {\kern 1pt} {\kern 1pt} \frac{h}{2}}} {\int_{{{\kern 1pt} {\kern 1pt} {\kern 1pt} 0}}^{{{\kern 1pt} {\kern 1pt} {\kern 1pt} l}} {{\kern 1pt} zQ_{11} \frac{\partial }{\partial x}\left( {\frac{{\partial u_{0} }}{\partial x} + \frac{1}{2}\left( {\frac{{\partial w_{0} }}{\partial x}} \right)^{2} + z\frac{{\partial \phi_{x} }}{\partial x} - z^{3} c_{1} \left( {\frac{{\partial \phi_{x} }}{\partial x} + \frac{{\partial^{2} w_{0} }}{{\partial x^{2} }}} \right)} \right)\delta \phi_{x} } } dxdzdt}\\ & \quad - b\int_{{{\kern 1pt} {\kern 1pt} {\kern 1pt} t_{1} }}^{{{\kern 1pt} {\kern 1pt} {\kern 1pt} t_{2} }} {\int_{{{\kern 1pt} {\kern 1pt} {\kern 1pt} - \frac{h}{2}}}^{{{\kern 1pt} {\kern 1pt} {\kern 1pt} \frac{h}{2}}} {\int_{{{\kern 1pt} {\kern 1pt} {\kern 1pt} 0}}^{{{\kern 1pt} {\kern 1pt} {\kern 1pt} l}} {{\kern 1pt} z^{3} c_{1} Q_{11} \frac{\partial }{\partial x}\left( {\frac{{\partial u_{0} }}{\partial x} + \frac{1}{2}\left( {\frac{{\partial w_{0} }}{\partial x}} \right)^{2} + z\frac{{\partial \phi_{x} }}{\partial x} - z^{3} c_{1} \left( {\frac{{\partial \phi_{x} }}{\partial x} + \frac{{\partial^{2} w_{0} }}{{\partial x^{2} }}} \right)} \right)} } \delta \phi_{x} dxdzdt}\\ &  \quad + b\int_{{{\kern 1pt} {\kern 1pt} {\kern 1pt} t_{1} }}^{{{\kern 1pt} {\kern 1pt} {\kern 1pt} t_{2} }} {\int_{{{\kern 1pt} {\kern 1pt} {\kern 1pt} - \frac{h}{2}}}^{{{\kern 1pt} {\kern 1pt} {\kern 1pt} \frac{h}{2}}} {\int_{{{\kern 1pt} {\kern 1pt} {\kern 1pt} 0}}^{{{\kern 1pt} {\kern 1pt} {\kern 1pt} l}} {{\kern 1pt} z^{3} c_{1} Q_{11} \frac{{\partial^{2} }}{{\partial x^{2} }}\left( {\frac{{\partial u_{0} }}{\partial x} + \frac{1}{2}\left( {\frac{{\partial w_{0} }}{\partial x}} \right)^{2} + z\frac{{\partial \phi_{x} }}{\partial x} - z^{3} c_{1} \left( {\frac{{\partial \phi_{x} }}{\partial x} + \frac{{\partial^{2} w_{0} }}{{\partial x^{2} }}} \right)} \right)} } \delta w_{0} dxdzdt} \\ & = b\int_{{{\kern 1pt} {\kern 1pt} {\kern 1pt} t_{1} }}^{{{\kern 1pt} {\kern 1pt} {\kern 1pt} t_{2} }} {\int_{{{\kern 1pt} {\kern 1pt} {\kern 1pt} - \frac{h}{2}}}^{{{\kern 1pt} {\kern 1pt} {\kern 1pt} \frac{h}{2}}} {\int_{{{\kern 1pt} {\kern 1pt} {\kern 1pt} 0}}^{{{\kern 1pt} {\kern 1pt} {\kern 1pt} l}} {{\kern 1pt} Q_{11} \frac{\partial }{\partial x}\left( {\frac{{\partial u_{0} }}{\partial x} + \frac{1}{2}\left( {\frac{{\partial w_{0} }}{\partial x}} \right)^{2} } \right)\delta u_{0} dx} } dzdt} \\ & \quad + b\int_{{{\kern 1pt} {\kern 1pt} {\kern 1pt} t_{1} }}^{{{\kern 1pt} {\kern 1pt} {\kern 1pt} t_{2} }} {\int_{{{\kern 1pt} {\kern 1pt} {\kern 1pt} - \frac{h}{2}}}^{{{\kern 1pt} {\kern 1pt} {\kern 1pt} \frac{h}{2}}} {\int_{{{\kern 1pt} {\kern 1pt} {\kern 1pt} 0}}^{{{\kern 1pt} {\kern 1pt} {\kern 1pt} l}} {{\kern 1pt} Q_{11} \frac{\partial }{\partial x}\left( {\frac{{\partial u_{0} }}{\partial x} + \frac{1}{2}\left( {\frac{{\partial w_{0} }}{\partial x}} \right)^{2} } \right)\frac{{\partial w_{0} }}{\partial x}\delta w_{0} } } dxdzdt} \\ & \quad  + b\int_{{{\kern 1pt} {\kern 1pt} {\kern 1pt} t_{1} }}^{{{\kern 1pt} {\kern 1pt} {\kern 1pt} t_{2} }} {\int_{{{\kern 1pt} {\kern 1pt} {\kern 1pt} - \frac{h}{2}}}^{{{\kern 1pt} {\kern 1pt} {\kern 1pt} \frac{h}{2}}} {\int_{{{\kern 1pt} {\kern 1pt} {\kern 1pt} 0}}^{{{\kern 1pt} {\kern 1pt} {\kern 1pt} l}} {{\kern 1pt} Q_{11} \left( {\frac{{\partial u_{0} }}{\partial x} + \frac{1}{2}\left( {\frac{{\partial w_{0} }}{\partial x}} \right)^{2} + z\frac{{\partial \phi_{x} }}{\partial x} - z^{3} c_{1} \left( {\frac{{\partial \phi_{x} }}{\partial x} + \frac{{\partial^{2} w_{0} }}{{\partial x^{2} }}} \right)} \right)\frac{{\partial^{2} w_{0} }}{{\partial x^{2} }}} } \delta w_{0} dxdzdt}  \\ & \quad  {\kern 1pt} {\kern 1pt} + b\int_{{{\kern 1pt} {\kern 1pt} {\kern 1pt} t_{1} }}^{{{\kern 1pt} {\kern 1pt} {\kern 1pt} t_{2} }} {\int_{{{\kern 1pt} {\kern 1pt} {\kern 1pt} - \frac{h}{2}}}^{{{\kern 1pt} {\kern 1pt} {\kern 1pt} \frac{h}{2}}} {\int_{{{\kern 1pt} {\kern 1pt} {\kern 1pt} 0}}^{{{\kern 1pt} {\kern 1pt} {\kern 1pt} l}} {{\kern 1pt} zQ_{11} \frac{\partial }{\partial x}\left( {z\frac{{\partial \phi_{x} }}{\partial x} - z^{3} c_{1} \left( {\frac{{\partial \phi_{x} }}{\partial x} + \frac{{\partial^{2} w_{0} }}{{\partial x^{2} }}} \right)} \right)\delta \phi_{x} } } dxdzdt}\\ & \quad  - b\int_{{{\kern 1pt} {\kern 1pt} {\kern 1pt} t_{1} }}^{{{\kern 1pt} {\kern 1pt} {\kern 1pt} t_{2} }} {\int_{{{\kern 1pt} {\kern 1pt} {\kern 1pt} - \frac{h}{2}}}^{{{\kern 1pt} {\kern 1pt} {\kern 1pt} \frac{h}{2}}} {\int_{{{\kern 1pt} {\kern 1pt} {\kern 1pt} 0}}^{{{\kern 1pt} {\kern 1pt} {\kern 1pt} l}} {{\kern 1pt} z^{3} c_{1} Q_{11} \frac{\partial }{\partial x}\left( {z\frac{{\partial \phi_{x} }}{\partial x} - z^{3} c_{1} \left( {\frac{{\partial \phi_{x} }}{\partial x} + \frac{{\partial^{2} w_{0} }}{{\partial x^{2} }}} \right)} \right)} } \delta \phi_{x} dxdzdt}  \\ & 
\quad + b\int_{{{\kern 1pt} {\kern 1pt} {\kern 1pt} t_{1} }}^{{{\kern 1pt} {\kern 1pt} {\kern 1pt} t_{2} }} {\int_{{{\kern 1pt} {\kern 1pt} {\kern 1pt} - \frac{h}{2}}}^{{{\kern 1pt} {\kern 1pt} {\kern 1pt} \frac{h}{2}}} {\int_{{{\kern 1pt} {\kern 1pt} {\kern 1pt} 0}}^{{{\kern 1pt} {\kern 1pt} {\kern 1pt} l}} {{\kern 1pt} z^{3} c_{1} Q_{11} \frac{{\partial^{2} }}{{\partial x^{2} }}\left( {z\frac{{\partial \phi_{x} }}{\partial x} - z^{3} c_{1} \left( {\frac{{\partial \phi_{x} }}{\partial x} + \frac{{\partial^{2} w_{0} }}{{\partial x^{2} }}} \right)} \right)} } \delta w_{0} dxdzdt} , \end{aligned}$$

7d $$ \begin{aligned} & L_{4}^{ * * } = - b\int_{{{\kern 1pt} {\kern 1pt} {\kern 1pt} t_{1} }}^{{{\kern 1pt} {\kern 1pt} {\kern 1pt} t_{2} }} {\int_{{{\kern 1pt} {\kern 1pt} {\kern 1pt} - \frac{h}{2}}}^{{{\kern 1pt} {\kern 1pt} {\kern 1pt} \frac{h}{2}}} {\int_{{{\kern 1pt} {\kern 1pt} {\kern 1pt} 0}}^{{{\kern 1pt} {\kern 1pt} {\kern 1pt} l}} {{\kern 1pt} Q_{13} \delta \left( {1 - 3c_{1} z^{2} } \right)\left( {\frac{{\partial w_{0} }}{\partial x} + \phi_{x} } \right)\varepsilon_{13} } } dxdzdt} \\ &= - b\int_{{{\kern 1pt} {\kern 1pt} {\kern 1pt} t_{1} }}^{{{\kern 1pt} {\kern 1pt} {\kern 1pt} t_{2} }} {\int_{{{\kern 1pt} {\kern 1pt} {\kern 1pt} - \frac{h}{2}}}^{{{\kern 1pt} {\kern 1pt} {\kern 1pt} \frac{h}{2}}} {\int_{{{\kern 1pt} {\kern 1pt} {\kern 1pt} 0}}^{{{\kern 1pt} {\kern 1pt} {\kern 1pt} l}} {{\kern 1pt} Q_{13} \left( {1 - 3c_{1} z^{2} } \right)\varepsilon_{13} } \frac{{\partial \delta w_{0} }}{\partial x}} dxdzdt} - b\int_{{{\kern 1pt} {\kern 1pt} {\kern 1pt} t_{1} }}^{{{\kern 1pt} {\kern 1pt} {\kern 1pt} t_{2} }} {\int_{{{\kern 1pt} {\kern 1pt} {\kern 1pt} - \frac{h}{2}}}^{{{\kern 1pt} {\kern 1pt} {\kern 1pt} \frac{h}{2}}} {\int_{{{\kern 1pt} {\kern 1pt} {\kern 1pt} 0}}^{{{\kern 1pt} {\kern 1pt} {\kern 1pt} l}} {{\kern 1pt} Q_{13} \left( {1 - 3c_{1} z^{2} } \right)\varepsilon_{13} \delta \phi_{x} } } dxdzdt}\\ &= 0 + b\int_{{{\kern 1pt} {\kern 1pt} {\kern 1pt} t_{1} }}^{{{\kern 1pt} {\kern 1pt} {\kern 1pt} t_{2} }} {\int_{{{\kern 1pt} {\kern 1pt} {\kern 1pt} - \frac{h}{2}}}^{{{\kern 1pt} {\kern 1pt} {\kern 1pt} \frac{h}{2}}} {\int_{{{\kern 1pt} {\kern 1pt} {\kern 1pt} 0}}^{{{\kern 1pt} {\kern 1pt} {\kern 1pt} l}} {{\kern 1pt} Q_{13} \left( {1 - 3c_{1} z^{2} } \right)\frac{{\partial \varepsilon_{13} }}{\partial x}} } \delta w_{0} dxdzdt} - b\int_{{{\kern 1pt} {\kern 1pt} {\kern 1pt} t_{1} }}^{{{\kern 1pt} {\kern 1pt} {\kern 1pt} t_{2} }} {\int_{{{\kern 1pt} {\kern 1pt} {\kern 1pt} - \frac{h}{2}}}^{{{\kern 1pt} {\kern 1pt} {\kern 1pt} \frac{h}{2}}} {\int_{{{\kern 1pt} {\kern 1pt} {\kern 1pt} 0}}^{{{\kern 1pt} {\kern 1pt} {\kern 1pt} l}} {{\kern 1pt} Q_{13} \left( {1 - 3c_{1} z^{2} } \right)\varepsilon_{13} \delta \phi_{x} } } dxdzdt}\\  &  = b\int_{{{\kern 1pt} {\kern 1pt} {\kern 1pt} t_{1} }}^{{{\kern 1pt} {\kern 1pt} {\kern 1pt} t_{2} }} {\int_{{{\kern 1pt} {\kern 1pt} {\kern 1pt} - \frac{h}{2}}}^{{{\kern 1pt} {\kern 1pt} {\kern 1pt} \frac{h}{2}}} {\int_{{{\kern 1pt} {\kern 1pt} {\kern 1pt} 0}}^{{{\kern 1pt} {\kern 1pt} {\kern 1pt} l}} {{\kern 1pt} Q_{13} \left( {1 - 3c_{1} z^{2} } \right)^{2} \left( {\frac{{\partial^{2} w_{0} }}{\partial x} + \frac{{\partial \phi_{x} }}{\partial x}} \right)} } \delta w_{0} dxdzdt}\\ & \quad - b\int_{{{\kern 1pt} {\kern 1pt} {\kern 1pt} t_{1} }}^{{{\kern 1pt} {\kern 1pt} {\kern 1pt} t_{2} }} {\int_{{{\kern 1pt} {\kern 1pt} {\kern 1pt} - \frac{h}{2}}}^{{{\kern 1pt} {\kern 1pt} {\kern 1pt} \frac{h}{2}}} {\int_{{{\kern 1pt} {\kern 1pt} {\kern 1pt} 0}}^{{{\kern 1pt} {\kern 1pt} {\kern 1pt} l}} {{\kern 1pt} Q_{13} \left( {1 - 3c_{1} z^{2} } \right)^{2} \left( {\frac{{\partial w_{0} }}{\partial x} + \phi_{x} } \right)\delta \phi_{x} } } dxdzdt} , \end{aligned}$$

Therefore, the equations of motion for the beam subject to external excitation is derived as,

8a $$ A_{11} \frac{{\partial^{2} u_{0} }}{{\partial x^{2} }} + A_{11} \frac{{\partial w_{0} }}{\partial x}\frac{{\partial^{2} w_{0} }}{{\partial x^{2} }} - I_{0} \frac{{d^{2} l}}{{dt^{2} }} - I_{0} \frac{{d^{2} u_{0} }}{{dt^{2} }} = 0, $$

$$ \left( { - A_{55} + 6D_{55} c_{1} - 9F_{55} c_{1}^{2} } \right)\phi_{x} + \left( {D_{11} - 2F_{11} c_{1} + H_{11} c_{1}^{2} } \right)\frac{{\partial^{2} \phi_{x} }}{{\partial x^{2} }} $$

$$ + \left( { - A_{55} + 6D_{55} c_{1} - 9F_{55} c_{1}^{2} } \right)\frac{{\partial w_{0} }}{\partial x} + \left( { - F_{11} c_{1} + H_{11} c_{1}^{2} } \right)\frac{{\partial^{3} w_{0} }}{{\partial x^{3} }} $$

8b $$ - K_{2} \frac{{d^{2} \phi_{x} }}{{dt^{2} }} + c_{1} J_{4} \frac{\partial }{\partial x}\left( {\frac{{d^{2} w_{0} }}{{dt^{2} }}} \right) = 0, $$

$$ A_{11} \frac{{\partial w_{0} }}{\partial x}\frac{{\partial^{2} u_{0} }}{{\partial x^{2} }} + A_{11} \frac{{\partial u_{0} }}{\partial x}\frac{{\partial^{2} w_{0} }}{{\partial x^{2} }} + \frac{3}{2}A_{11} \left( {\frac{{\partial w_{0} }}{\partial x}} \right)^{2} \frac{{\partial^{2} w_{0} }}{{\partial x^{2} }} + c_{1} \left( {F_{11} - c_{1} H_{11} } \right)\frac{{\partial^{3} \phi_{x} }}{{\partial x^{3} }} - c_{1}^{2} H_{11} \frac{{\partial^{4} w_{0} }}{{\partial x^{4} }} - \left( {\frac{{4q_{d} \gamma }}{{VM_{\infty } }} + c} \right)\frac{{dw_{0} }}{dt} $$

$$ + \left( {A_{55} - 6c_{1} D_{55} + 9c_{1}^{2} F_{55} } \right)\frac{{\partial \phi_{x} }}{\partial x} + \left( {A_{55} - 6c_{1} D_{55} + 9c_{1}^{2} F_{55} } \right)\frac{{\partial^{2} w_{0} }}{{\partial^{2} x}} $$

8c $$ - I_{0} \frac{{d^{2} w_{0} }}{{dt^{2} }} + c_{1}^{2} I_{6} \frac{{\partial^{2} }}{{\partial x^{2} }}\left( {\frac{{d^{2} w_{0} }}{{dt^{2} }}} \right) - c_{1} J_{4} \frac{\partial }{\partial x}\left( {\frac{{d^{2} \phi_{x} }}{{dt^{2} }}} \right) = 0, $$

where $A_{11}$, $K_{2}$, $D_{11}$, $F_{11}$, $H_{11}$, $A_{55}$, $D_{55}$,$F_{55}$, $I_{0}$, $I_{4}$, and $I_{6}$ are provided in the Supplementary Appendix, and

9 $$ J_{i} = I_{i} - I_{i + 2} c_{1} ,\quad K_{2} = \left( {I_{2} - 2I_{4} c_{1} + I_{6} c_{1}^{2} } \right), $$

and $i = \left( {0,1,2,...,6} \right)$; $\overline{Q}_{ij}^{\left( 1 \right)}$, $\overline{Q}_{ij}^{\left( 2 \right)}$ and $\overline{Q}_{ij}^{\left( 3 \right)}$ are the stiffness coefficients for the lower layer, the middle layer, and the upper layer of the beam, and $\rho^{(1)}$, $\rho^{(2)}$ and $\rho^{(3)}$ are the densities for the corresponding layers.

Based on Eq. (8a) and Eq. (8b), it is obtained that,

10a $$ \frac{{\partial u_{0} }}{\partial x} = - \frac{1}{2}\left( {\frac{{\partial w_{0} }}{\partial x}} \right)^{2} + \frac{1}{2l}\int_{{{\kern 1pt} {\kern 1pt} {\kern 1pt} 0}}^{{{\kern 1pt} {\kern 1pt} l}} {\left( {\frac{{\partial w_{0} }}{\partial x}} \right)^{2} dx} + \frac{{I_{0} }}{{A_{11} }}\frac{{d^{2} x}}{{dt^{2} }}\left( {x - \frac{l}{2}} \right), $$

10b $$ \phi_{x} = - \frac{{\partial w_{0} }}{\partial x} + \frac{{F_{11} c_{1} - D_{11} }}{{\left( {A_{55} - 6D_{55} c_{1} + 9F_{55} c_{1}^{2} } \right)}}\frac{{\partial^{3} w_{0} }}{{\partial^{3} x}}. $$

Substitute Eq. (10) into Eq. (8c), it can be obtained as follows,

$$ - I_{0} \frac{{d^{2} w_{0} }}{{dt^{2} }} + c_{1} I_{4} \frac{{\partial^{2} }}{{\partial x^{2} }}\left( {\frac{{d^{2} w_{0} }}{{dt^{2} }}} \right) - c_{1} J_{4} \frac{{F_{11} c_{1} - D_{11} }}{{\left( {A_{55} - 6D_{55} c_{1} + 9F_{55} c_{1}^{2} } \right)}}\frac{{\partial^{4} }}{{\partial^{4} x}}\left( {\frac{{d^{2} w_{0} }}{{dt^{2} }}} \right) $$

$$ - c\frac{{dw_{0} }}{dt} + \frac{{A_{11} }}{{2l_{0} }}\frac{{\partial^{2} w_{0} }}{{\partial x^{2} }}\left[ {\int_{{{\kern 1pt} {\kern 1pt} {\kern 1pt} 0}}^{{{\kern 1pt} {\kern 1pt} {\kern 1pt} l}} {\left( {\frac{{\partial w_{0} }}{\partial x}} \right)^{2} } dx} \right] - D_{11} \frac{{\partial^{4} w_{0} }}{{\partial^{4} x}} $$

11 $$ + c_{1} \left( {F_{11} - c_{1} H_{11} } \right)\frac{{F_{11} c_{1} - D_{11} }}{{\left( {A_{55} - 6D_{55} c_{1} + 9F_{55} c_{1}^{2} } \right)}}\frac{{\partial^{6} w_{0} }}{{\partial^{6} x}} + q = 0. $$

## Non-dimensionalization

To be concise^2,14^, introduce the non-dimensional variables below into Eq. (11),

$$ \overline{t} = \sqrt {\frac{{Q_{11}^{\left( 2 \right)} I}}{{I_{0} bl^{4} }}} t = \tau t,\quad \overline{x} = \frac{x}{l},\quad \overline{l} = \frac{1}{h}l,\quad \overline{w}_{0} = \frac{{w_{0} }}{h}, $$

12 $$ \frac{{d\overline{w}_{0} }}{{d\overline{t}}} = \frac{1}{\tau h}\frac{{dw_{0} }}{dt},\quad \frac{{d^{2} \overline{w}_{0} }}{{d\overline{t}^{2} }} = \frac{1}{{\tau^{2} h}}\frac{{d^{2} w_{0} }}{{dt^{2} }},\quad \overline{c} = {c \mathord{\left/ {\vphantom {c {\left( {\frac{{Q_{11}^{\left( 2 \right)} }}{h\tau }} \right)}}} \right. \kern-0pt} {\left( {\frac{{Q_{11}^{\left( 2 \right)} }}{h\tau }} \right)}}, $$

where,

$$ I = \int\limits_{\Omega } {z^{2} dA_{zy} } = \frac{{bh^{3} }}{12},\quad I_{0} = \sum\limits_{k = 1}^{3} {\int_{{z_{k - 1} }}^{{z_{k} }} {\rho^{(k)} dz} } . $$

Substitute the non-dimensional variables above into Eq. (11), it is derived,

$$ - A\frac{{d^{2} \overline{w}_{0} }}{{d\overline{t}^{2} }} + B\frac{{\partial^{2} }}{{\partial \overline{x}^{2} }}\left( {\frac{{d^{2} \overline{w}_{0} }}{{d\overline{t}^{2} }}} \right) - C\frac{{\partial^{4} }}{{\partial \overline{x}^{4} }}\left( {\frac{{d^{2} \overline{w}_{0} }}{{d\overline{t}^{2} }}} \right) - D\frac{{d\overline{w}_{0} }}{{d\overline{t}}} $$

13 $$ + F\frac{{\partial^{2} \overline{w}_{0} }}{{\partial \overline{x}^{2} }}\left[ {\int_{0}^{1} {\left( {\frac{{\partial \overline{w}_{0} }}{{\partial \overline{x}}}} \right)^{2} dx} } \right] - G\frac{{\partial^{4} \overline{w}_{0} }}{{\partial \overline{x}^{4} }} + H\frac{{\partial^{6} \overline{w}_{0} }}{{\partial \overline{x}^{6} }} + \overline{q} = 0, $$

where, $A$, $B$, $C$, $D$, $E$, $F$, $G$, and $H$ are provided in the Supplementary Appendix. In the following sections, $\overline{w}_{1}$, $\overline{w}_{2}$, $\overline{t}$, and $\overline{q}$ will be substituted with $w_{1}$, $w_{2}$, $t$ and $q$ for convenience.

## Series solutions

$W_{0}$ is expanded in terms of comparison functions as follows,

14 $$ w_{0} = \sum\limits_{n = 1}^{\infty } {\phi_{n} \left( x \right)w_{n} \left( t \right)} , $$

According to the boundary conditions of the cantilever beam, $\phi_{n} \left( x \right)$ is given as follows,

$$ \phi_{n} \left( x \right) = \left[ {ch\left( {\lambda_{n} x} \right) - \cos \left( {\lambda_{n} x} \right)} \right] - \frac{{\left( {ch{\uplambda }_{n} + \cos {\uplambda }_{n} } \right)}}{{\left( {sh{\uplambda }_{n} + \sin {\uplambda }_{n} } \right)}}\left[ {sh\left( {\lambda_{n} x} \right) - \sin \left( {\lambda_{n} x} \right)} \right]. $$

where, $\lambda_{1}$ and $\lambda_{2}$ are given as 1.875 and 4.694 if a 2nd order Galerkin method is applied.

Substitute the series solution in Eq. (14) in the case of $n = 2$ into Eq. (13), Eq. (14) at a specified point P of the beam ($x = x_{{\text{P}}} = 0.75$) and the governing equation with the 2^nd^ order Galerkin method is obtained as,

15 $$ w_{{\text{P}}} = \sum\limits_{n = 1}^{2} {\phi_{n} \left( {x_{{\text{P}}} } \right)w_{n} \left( t \right) = 1.315382461} w_{1} \left( t \right) + 0.27008056w_{2} \left( t \right), $$

16 $$ \left\{ {\begin{array}{*{20}c} {\dot{w}_{1,1} = w_{1,2} } \\ {\dot{w}_{1,2} = T_{11} w_{1,2} + T_{12} w_{1,1} + T_{13} w_{2,2} + T_{14} w_{2,1} + T_{15} w_{1,1}^{.3} + T_{16} w_{1,1}^{.2} w_{2,1} + T_{17} w_{2,1}^{.2} w_{1,1} + T_{18} w_{2,1}^{.3} + T_{19} q} \\ {\dot{w}_{2,1} = w_{2,2} } \\ {\dot{w}_{2,2} = T_{21} w_{1,2} + T_{22} w_{1,1} + T_{23} w_{2,2} + T_{24} w_{2,1} + T_{25} w_{1,1}^{.3} + T_{26} w_{1,1}^{.2} w_{2,1} + T_{27} w_{2,1}^{.2} w_{1,1} + T_{28} w_{2,1}^{.3} + T_{29} q} \\ \end{array} } \right.{\kern 1pt} $$

where $T_{1i}$, $T_{2i}$ ($i = 1,2,...,9$), are provided in the Supplementary Appendix.

## Chaotic motion

In this section, the vibration of the point P on the laminated composite cantilever beam is studied with the employment of the software Matlab. Through the numerical simulations, a chaotic vibration is discovered.

**Figure 2:**
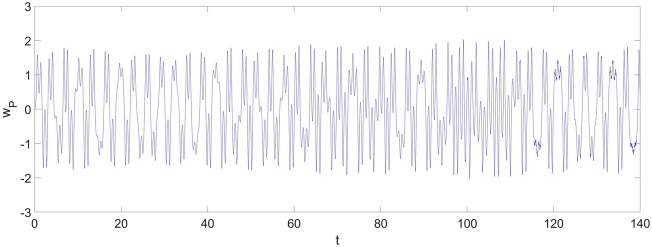
The vibration of the cantilever beam at $x_{{\text{P}}} = 0.75$ without applying the control strategy.

Given the geometric parameters,

17 $$ l_{0} = 0.5\;{\text{m,}}\quad b = 0.02\;{\text{m}},\quad \, h = 0.01\;{\text{m}} $$

and the excitation,

18 $$ q = 5500{\text{sin}} \, \left( {{20}\pi t} \right)\;{\text{Pa,}}\quad c = 0.01\;{\text{N}}/(({\text{m}}/{\text{s}}){\text{m}}^{{2}} ) \, $$

and the nondimensional initial conditions,

19 $$ w_{1} \left( 0 \right) = 0,\quad \frac{{dw_{1} \left( 0 \right)}}{dt} = 0,\quad w_{2} \left( 0 \right) = 0,\quad \frac{{dw_{2} \left( 0 \right)}}{dt} = 0, $$

the nonlinear vibration derived from Eqs. (15, 16) at the selected point are shown in Fig. 2.

**Figure 3:**
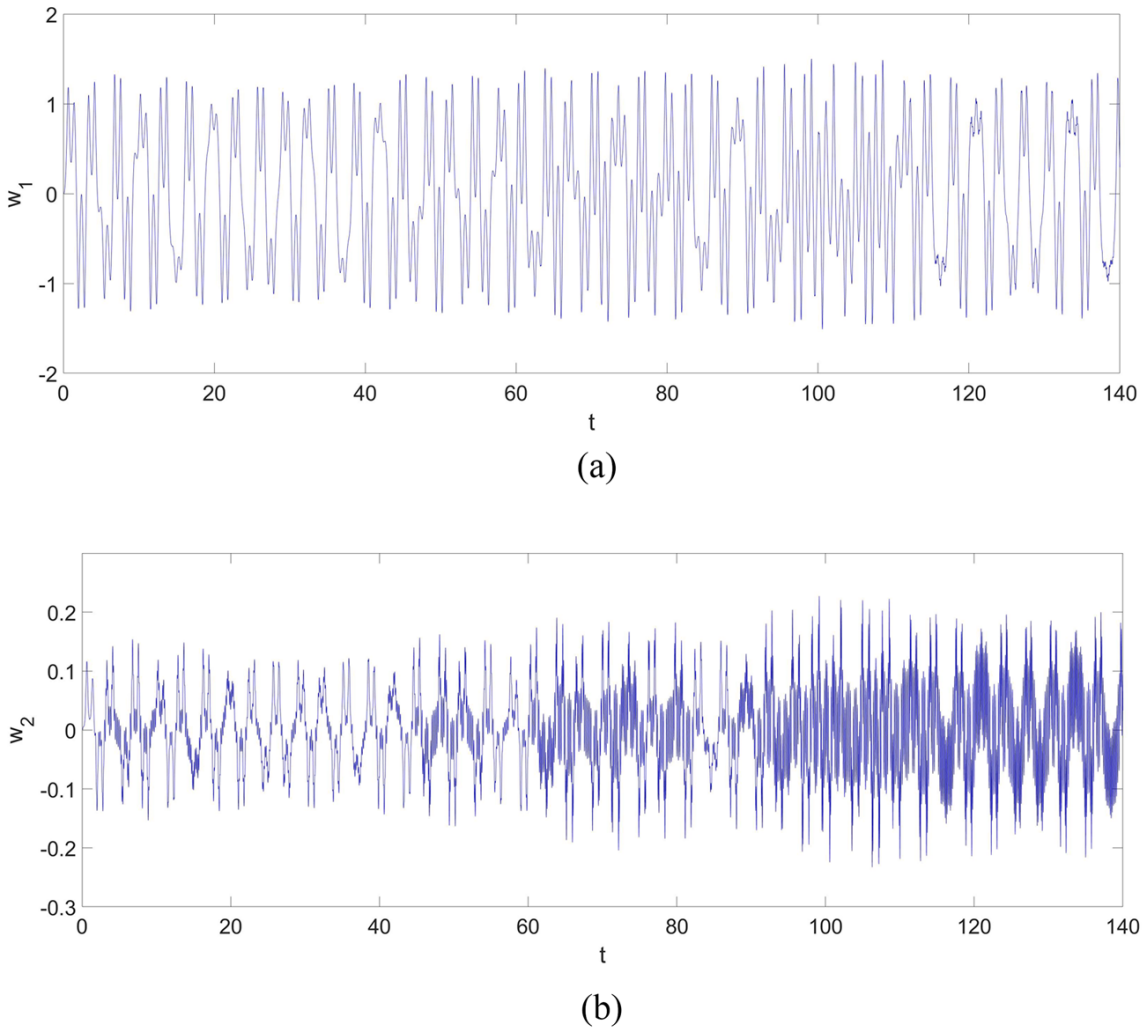
The vibration of the cantilever beam for the first two vibration mode: (**a**) $w_{1}$; (**b**) $w_{2}$.

From Fig. 2, one can discover a chaotic vibration in the two-dimensional nonlinear dynamic system of the cantilever beam. The chaotic vibration features a large amplitude increasing up to 2, which means the maximum amplitude of the chaotic response can be twice the thickness of the cantilever beam. The chaotic vibrations of $w_{1}$ and $w_{2}$ are given in Fig. 3.

From Fig. 3a, b, the maximum amplitude of $w_{1}$ is around 1.5, and the maximum amplitude of $w_{2}$ is around 0.2. Therefore, the contribution of $w_{2}$ cannot be neglected, and a multi-dimensional nonlinear dynamic system of the cantilever beam should be considered if an accurate vibration estimation of the beam is required.

In response to the large-amplitude chaotic motion shown in Fig. 2, a modified control strategy based on the FSMC is needed to stabilize and reduce the nonlinear vibration.

## Control strategy

In the previous works^29,33^, the target system to be synchronized, can be generalized as,

20 $$ \left\{ {\begin{array}{*{20}c} {\dot{y}_{j} = y_{j + 1} {\kern 1pt} {\kern 1pt} {\kern 1pt} {\kern 1pt} {\kern 1pt} {\kern 1pt} {\kern 1pt} {\kern 1pt} } \\ {\dot{y}_{n} = f\left( {{\mathbf{Y}},t} \right) + d\left( {{\mathbf{Y}},t} \right) + u} \\ {y^{o} = y_{\kappa } } \\ \end{array} } \right.{\kern 1pt} , $$

and the corresponding system as a reference is,

21 $$ \left\{ {\begin{array}{*{20}c} {\dot{x}_{j} = x_{j + 1} {\kern 1pt} {\kern 1pt} {\kern 1pt} {\kern 1pt} {\kern 1pt} {\kern 1pt} {\kern 1pt} {\kern 1pt} } \\ {\dot{x}_{n} = g\left( {{\mathbf{X}},t} \right)} \\ {x_{\kappa }^{o} = x_{\kappa } } \\ \end{array} } \right.{\kern 1pt} , $$

where $1 \le j \le n - 1$, ${\text{Y}} = \left[ {y_{1} y_{2} ... y_{n} } \right]^{T} \in {\text{R}}^{n}$, ${\text{X}} = \left[ {x_{1} x_{2} ... x_{n} } \right]^{T} \in {\text{R}}^{n}$, $f\left( {{\mathbf{Y}},t} \right)$ is the specified expression of $\dot{y}_{n}$, $d\left( {{\mathbf{Y}},t} \right)$ represents the uncertain external disturbance applied to the system and is defined as $\left| {d\left( {{\text{Y}},t} \right)} \right| \le B_{boundary} \in {\text{R}}^{ + }$, $u \in {\text{R}}$ denotes the control input, ${\mathbf{Y}}^{o} = \left[ {x_{1}^{o} {\kern 1pt} {\kern 1pt} {\kern 1pt} x_{2}^{o} {\kern 1pt} {\kern 1pt} {\kern 1pt} ...{\kern 1pt} {\kern 1pt} {\kern 1pt} x_{\kappa }^{o} } \right]^{T}$ ($\kappa \le j$) is the output selected in ${\mathbf{Y}}$, and ${\mathbf{X}}^{o} = \left[ {x_{1}^{o} {\kern 1pt} {\kern 1pt} {\kern 1pt} x_{2}^{o} {\kern 1pt} {\kern 1pt} {\kern 1pt} ...{\kern 1pt} {\kern 1pt} {\kern 1pt} x_{\kappa }^{o} } \right]^{T}$ represents the reference vibration corresponding to ${\mathbf{Y}}^{o}$.

However, it should be noticed that: the control strategy shown in Eqs. (20, 21) will not be available for a multi-dimensional nonlinear dynamic system of a continuum beam structure, such as the one in Eq. (16). The numerical simulation shown in Fig. 3 in the previous section, along with the published works^2,14^, demonstrates that a multi-dimensional nonlinear dynamic system of a continuum structure such as a cantilever beam is necessary for chaotic vibration analysis. Therefore, a modified control strategy based on the existing FSMC has been proposed to control the chaotic vibration of the multi-dimensional nonlinear dynamic system of a continuum cantilever beam structure.

Corresponding to a nonlinear equation in the form below (such as Eq. 13)

22 $$ \ddot{w} = \Phi \left( {w,\dot{w},t} \right), $$

if $U$ represents the control input and $\Delta F\left( {w,\dot{w}} \right)$ is given as the unknown external disturbance imposed on the cantilever beam, the equation in Eq. (22) will be,

23 $$ \ddot{w} = \Phi \left( {w,\dot{w},t} \right) + U + \Delta F\left( {w,\dot{w}} \right). $$

If the *n*th-order Galerkin method is implemented in discretizing the governing equation in Eq. (23), a series of second-order ordinary differential equations including $U$ and $\Delta F\left( {w,\dot{w}} \right)$ can be obtained in the following,

24 $$ \left\{ {\begin{array}{*{20}c} {\dot{w}_{1,1} = w_{1,2} } \\ {\dot{w}_{1,2} = \phi_{1} \left( {{\mathbf{W}},t} \right) + u_{1} + \Delta f_{1} \left( {{\mathbf{W}},t} \right)} \\ {\dot{w}_{2,1} = w_{2,2} } \\ {\dot{w}_{2,2} = \phi_{2} \left( {{\mathbf{W}},t} \right) + u_{2} + \Delta f_{2} \left( {{\mathbf{W}},t} \right)} \\ \vdots \\ {\dot{w}_{i,1} = w_{i,2} } \\ {\dot{w}_{i,2} = \phi_{i} \left( {{\mathbf{W}},t} \right) + u_{i} + \Delta f_{i} \left( {{\mathbf{W}},t} \right)} \\ \vdots \\ {\dot{w}_{n,1} = w_{n,2} } \\ {\dot{w}_{n,2} = \phi_{n} \left( {{\mathbf{W}},t} \right) + u_{n} + \Delta f_{i} \left( {{\mathbf{W}},t} \right)} \\ \end{array} } \right.{\kern 1pt} , $$

where, $\phi_{i} \left( {{\mathbf{W}},t} \right)$, $u_{i}$, and $\Delta f_{i} \left( {{\mathbf{W}},t} \right)$ are the specific form of $\Phi \left( {w,\dot{w},t} \right)$, $U$, and $\Delta F\left( {w,\dot{w}} \right)$ after applying the Galerkin method.

Then, the column vector ${\mathbf{W}}$ in Eq. (24) can be obtained below,

$$ {\mathbf{W}} = \left[ {w_{1,1} {\kern 1pt} {\kern 1pt} {\kern 1pt} w_{1,2} {\kern 1pt} {\kern 1pt} w_{2,1} {\kern 1pt} {\kern 1pt} {\kern 1pt} w_{2,2} {\kern 1pt} {\kern 1pt} {\kern 1pt} {\kern 1pt} \cdots {\kern 1pt} {\kern 1pt} {\kern 1pt} {\kern 1pt} {\kern 1pt} w_{i,1} {\kern 1pt} {\kern 1pt} {\kern 1pt} w_{i,2} {\kern 1pt} {\kern 1pt} {\kern 1pt} {\kern 1pt} \cdots {\kern 1pt} {\kern 1pt} {\kern 1pt} {\kern 1pt} {\kern 1pt} w_{n,1} {\kern 1pt} {\kern 1pt} {\kern 1pt} w_{n,2} } \right]^{T} . $$

According to Eq. (13) and Eq. (24), the nonlinear response of the specified point *w*_P_ is expressed as

25 $$ w_{{\text{P}}} = \sum\limits_{n = 1}^{\infty } {\phi_{n} \left( {x_{{\text{P}}} } \right)w_{n} \left( t \right)} , $$

where $x_{{\text{P}}}$ is the position of the specified point.

In the case of a desired reference vibration given below,

26 $$ w_{r} = \Psi \left( t \right), $$

$U$ is expressed as,

27 $$ U = U_{eq} - U_{r} , $$

where $U_{eq}$ and $U_{r}$ are provided below,

28 $$ U_{eq} = - \left( {\left( {\dot{w}_{p} - \dot{\Psi }} \right) + \kappa \left( {w_{p} - \Psi } \right)} \right),\;U_{r} = k_{fs} U_{fs} . $$

In Eq. (28), $\kappa$ is the control parameter governing the sliding surface, $k_{fs}$ is expressed as $\left| {\Delta F\left( {w,\dot{w}} \right)} \right| < k_{fs} \in R^{ + }$, and $U_{fs}$ is defined based on the fuzzy rule given in Table 1^31^.

**Table 1:** The fuzzy rule of $U_{fs}$.

$U_{fs}$		*U*_*eq*_						
		PB	PM	PS	ZE	NS	NM	NB
$\frac{{dU_{eq} }}{dt}$	PB	NB	NB	NB	NB	NM	NS	ZE
	PM	NB	NB	NB	NM	NS	ZE	PS
	PS	NB	NB	NM	NS	ZE	PS	PM
	ZE	NB	NM	NS	ZE	PS	PM	PB
	NS	NM	NS	ZE	PS	PM	PB	PB
	NM	NS	ZE	PS	PM	PB	PB	PB
	NB	ZE	PS	PM	PB	PB	PB	PB

**Figure 4:**
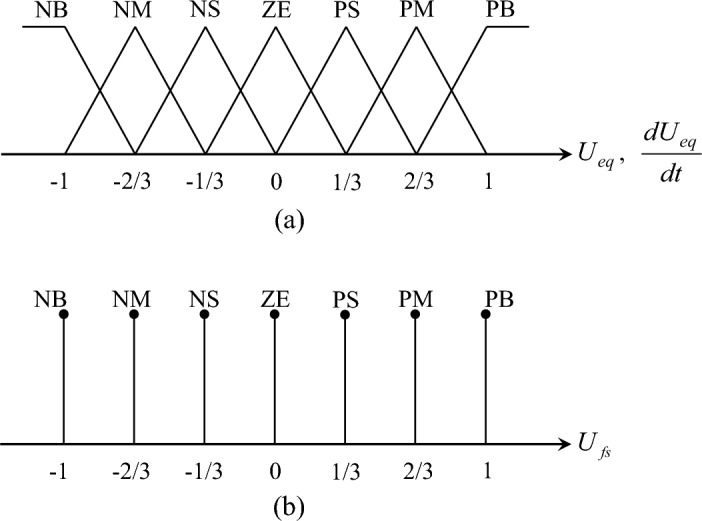
The membership functions of the input–output variables: (**a**) the membership functions of $U_{eq}$ and $\frac{{dU_{eq} }}{dt}$; (**b**) the membership function of $U_{fs}$.

In addition to the fuzzy rules provided in Table 1, the detailed membership functions of the input–output fuzzy variables, $U_{eq}$, $\frac{{dU_{eq} }}{dt}$, and $U_{fs}$ have been described in Fig. 4a, b respectively, based on the previous research^17,28,29,31^.

With the application of the control strategy in Eqs. (23–28), the nonlinear vibration control of the governing equation in Eq. (22) will be realized.

Take the cantilever beam governed by Eq. (13) as a case study. Implement the proposed modified control strategy and apply the control input given in Eq. (23), and the governing equation including the control input is obtained as follows,

$$ - A\frac{{d^{2} \overline{w}_{0} }}{{d\overline{t}^{2} }} + B\frac{{\partial^{2} }}{{\partial \overline{x}^{2} }}\left( {\frac{{d^{2} \overline{w}_{0} }}{{d\overline{t}^{2} }}} \right) - C\frac{{\partial^{4} }}{{\partial \overline{x}^{4} }}\left( {\frac{{d^{2} \overline{w}_{0} }}{{d\overline{t}^{2} }}} \right) - D\frac{{d\overline{w}_{0} }}{{d\overline{t}}} $$

29 $$ + F\frac{{\partial^{2} \overline{w}_{0} }}{{\partial \overline{x}^{2} }}\left[ {\int_{0}^{1} {\left( {\frac{{\partial \overline{w}_{0} }}{{\partial \overline{x}}}} \right)^{2} dx} } \right] - G\frac{{\partial^{4} \overline{w}_{0} }}{{\partial \overline{x}^{4} }} + H\frac{{\partial^{6} \overline{w}_{0} }}{{\partial \overline{x}^{6} }} + \overline{q} - U - \Delta F\left( {w,\dot{w}} \right) = 0. $$

Applying the second-order Galerkin method, Eq. (29) will become,

30 $$ \left\{ {\begin{array}{*{20}c} {\dot{w}_{1,1} = w_{1,2} } \\ \begin{gathered} \dot{w}_{1,2} = T_{11} w_{1,2} + T_{12} w_{1,1} + T_{13} w_{2,2} + T_{14} w_{2,1} + T_{15} w_{1,1}^{.3} \hfill \\ {\kern 1pt} {\kern 1pt} {\kern 1pt} {\kern 1pt} {\kern 1pt} {\kern 1pt} {\kern 1pt} {\kern 1pt} {\kern 1pt} {\kern 1pt} {\kern 1pt} {\kern 1pt} {\kern 1pt} {\kern 1pt} {\kern 1pt} {\kern 1pt} {\kern 1pt} {\kern 1pt} {\kern 1pt} {\kern 1pt} {\kern 1pt} {\kern 1pt} {\kern 1pt} {\kern 1pt} {\kern 1pt} {\kern 1pt} {\kern 1pt} {\kern 1pt} + T_{16} w_{1,1}^{.2} w_{2,1} + T_{17} w_{2,1}^{.2} w_{1,1} + T_{18} w_{2,1}^{.3} + T_{19} q + u_{1} + \Delta f_{1} \left( {{\mathbf{W}},t} \right) \hfill \\ \end{gathered} \\ {\dot{w}_{2,1} = w_{2,2} } \\ \begin{gathered} \dot{w}_{2,2} = T_{21} w_{1,2} + T_{22} w_{1,1} + T_{23} w_{2,2} + T_{24} w_{2,1} + T_{25} w_{1,1}^{.3} \hfill \\ {\kern 1pt} {\kern 1pt} {\kern 1pt} {\kern 1pt} {\kern 1pt} {\kern 1pt} {\kern 1pt} {\kern 1pt} {\kern 1pt} {\kern 1pt} {\kern 1pt} {\kern 1pt} {\kern 1pt} {\kern 1pt} {\kern 1pt} {\kern 1pt} {\kern 1pt} {\kern 1pt} {\kern 1pt} {\kern 1pt} {\kern 1pt} {\kern 1pt} {\kern 1pt} {\kern 1pt} {\kern 1pt} {\kern 1pt} {\kern 1pt} {\kern 1pt} + T_{26} w_{1,1}^{.2} w_{2,1} + T_{27} w_{2,1}^{.2} w_{1,1} + T_{28} w_{2,1}^{.3} + T_{29} q + u_{2} + \Delta f_{2} \left( {{\mathbf{W}},t} \right) \hfill \\ \end{gathered} \\ \end{array} } \right.{\kern 1pt} , $$

where, $u_{1}$ and $u_{2}$ are obtained through the second-order Galerkin method as follows,

$$u_{1} = 0.7849249756U \,\,\, u_{2} = 0.4319801434U.$$

## Vibration control

With the employment of Matlab, the control strategy proposed in the previous section will be applied in synchronizing the chaotic vibration of the cantilever beam at the selected point with a desired reference.

The control strategy is applied at *t* = 173, and the control parameters are given below,

31 $$ w_{r} = 1.4\sin \left( {1.9862t} \right),\quad \kappa = 0.1,\quad k_{fs} = 0.1,\quad \Delta F\left( {w,\dot{w}} \right) = 0.01\sin (w_{p} ). $$

**Figure 5:**
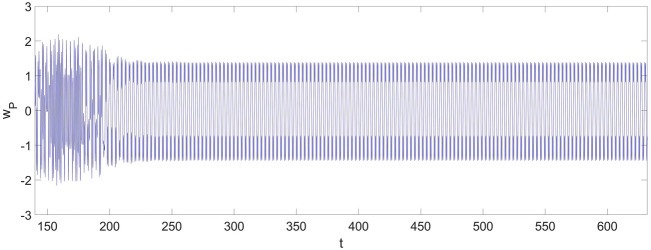
The vibration of the cantilever beam at $x_{{\text{P}}} = 0.75$ with the application of the control strategy.

**Figure 6:**
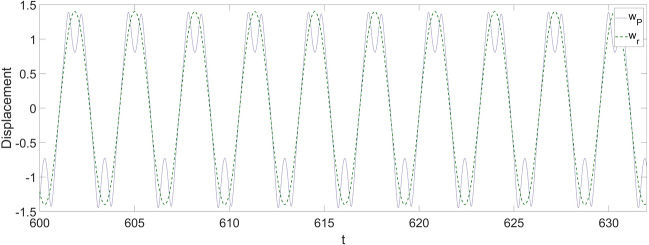
The comparison between the response at $x_{{\text{P}}} = 0.75$ and the response of the desired reference.

**Figure 7:**
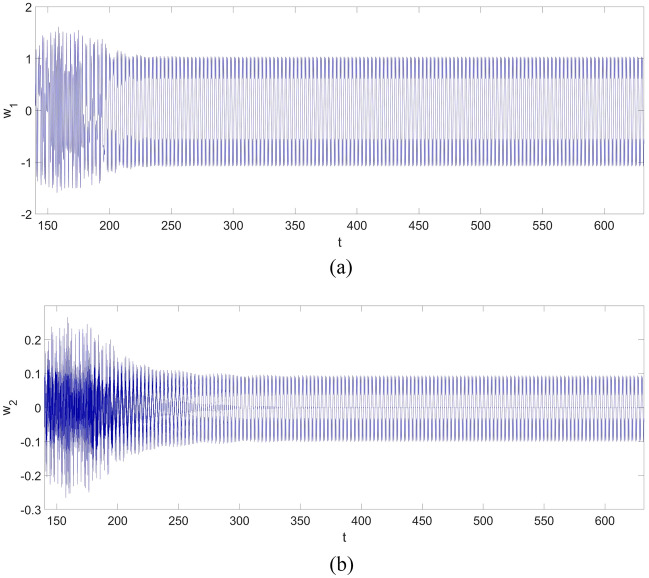
The vibration of the cantilever beam for the first two vibration modes with the application of the control strategy: (**a**) $w_{1}$; (**b**) $w_{2}$.

Following Eqs. (24, 26, 27), the vibrations of the system after the implementation of the proposed control strategy are shown in Figs. 5, 6, and 7.

From Fig. 5, the maximum amplitude of the vibration of the beam is significantly reduced by 30% from about 2 to 1.4, and the actual vibration at $x_{{\text{P}}} = 0.75$ on the beam will finally be stabilized and synchronized with the reference vibration. It should be noticed that the stabilization process takes about 50 non-dimensional time units before the response finally gets synchronized. In Fig. 6, a comparison is provided to further examine the implementation of the control strategy, and the response at $x_{{\text{P}}} = 0.75$ is well synchronized with the reference vibration, despite some slight discrepancies existing in the regions where the vibration of the beam reaches its amplitude.

Figure 7 shows the responses of the first two vibration modes. Clearly, both $w_{1}$ and $w_{2}$ finally become periodic motions with the implementation of the control strategy, and their amplitudes are reduced as well.

**Figure 8:**
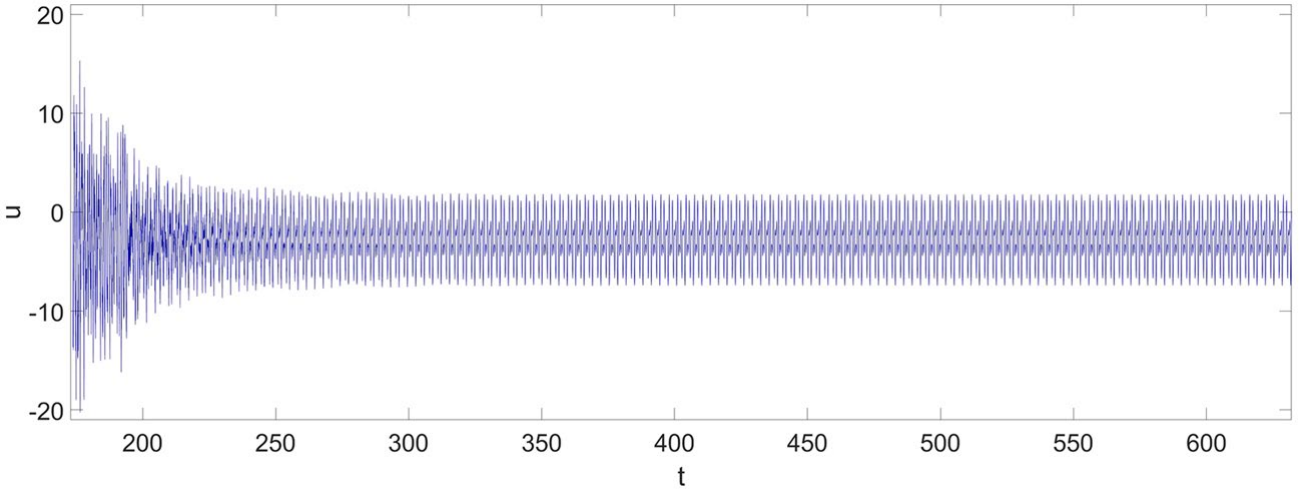
The control input.

Figure 8 shows the control input. The control input dramatically reaches to the highest value the moment the control strategy is applied, and its maximum value is about 20. In the stabilization process, which starts at *t* = 173 and ends at about *t* = 225, the control input gradually decreases, and it stops decreasing once the actual response at $x_{{\text{P}}} = 0.75$ is synchronized with the reference vibration. Compare the control inputs between the initial value and the value at the end of the stabilization, it can be learned: once the vibration of the beam is stabilized, only a small value of the control input is required to maintain the synchronization. Thus, the efficiency of the control strategy for vibration reduction is demonstrated.

## Conclusions

In this research, a control strategy modified based on the FSMC is implemented in the vibration control of a laminated composite beam considering the 3^rd^ order shearing effect. In the study of the chaotic vibration of the beam, it is discovered: a two-dimensional nonlinear dynamic system is necessary in the prediction of a cantilever beam. However, the FSMC is not originally established for such multi-dimensional systems. Therefore, the existing FSMC has been modified, and then applied in the nonlinear vibration control of the dynamic behavior of the cantilever beam. The numerical results feature both the effectiveness in the vibration control and the efficiency as shown in the control cost during the application process.

### Future development

To enhance the applicability and improve the control efforts of the proposed modified FSMC, the realization of the established control strategy involving sensors and actuators would be a promising research topic.

### Supplementary Information

Supplementary Information.

## Data Availability

The datasets used and analyzed during the current study are available from the corresponding author on reasonable request.
