# Supplementary material for: Chaotic vibration control of a composite cantilever beam
Source: Sci Rep. 2023 Oct 20;13:17946. doi: 10.1038/s41598-023-45113-3 (PMC10589266; doi:10.1038/s41598-023-45113-3)
Supplement: Supplementary file 1 — Supplementary Information. [file 41598_2023_45113_MOESM1_ESM.docx]

**Appendix**

, ,

$\left(I_{0},I_{1},I_{2},I_{3},I_{4},I_{5},I_{6}\right)=\sum_{k=1}^{3} \int_{z_{k-1}}^{z_{k}} \rho^{(k)}\left(\text{1,z}^{1}\text{,z}^{2}\text{,z}^{3}\text{,z}^{4}\text{,z}^{5}\text{,z}^{6}\right)\text{dz}$.

$A=\bar{I}_{0}$, , , , , , .

$T_{11}=U_{1}S_{1b}+U_{2}S_{2b}$,$T_{12}=U_{1}S_{1c}+U_{2}S_{2c}$,$T_{13}=U_{1}S_{1e}+U_{2}S_{2e}$,$T_{14}=U_{1}S_{1f}+U_{2}S_{2f}$, $T_{15}=U_{1}S_{1g}+U_{2}S_{2g}$,$T_{16}=U_{1}S_{1h}+U_{2}S_{2h}$,$T_{17}=U_{1}S_{1i}+U_{2}S_{2i}$,$T_{18}=U_{1}S_{1j}+U_{2}S_{2j}$,$T_{19}=U_{1}S_{1k}+U_{2}S_{2k}$;

$T_{21}=U_{3}S_{1b}+U_{4}S_{2b}$,$T_{22}=U_{3}S_{1c}+U_{4}S_{2c}$,$T_{23}=U_{3}S_{1e}+U_{4}S_{2e}$,$T_{24}=U_{3}S_{1f}+U_{4}S_{2f}$,

$T_{25}=U_{3}S_{1g}+U_{4}S_{2g}$,$T_{26}=U_{3}S_{1h}+U_{4}S_{2h}$,$T_{27}=U_{3}S_{1i}+U_{4}S_{2i}$,$T_{28}=U_{3}S_{1j}+U_{4}S_{2j}$, $T_{29}=U_{3}S_{1k}+U_{4}S_{2k}$;

$U_{1}=-\frac{S_{2d}}{S_{1a}S_{2d}-S_{1d}S_{2a}}$, $U_{2}=\frac{S_{1d}}{S_{1a}S_{2d}-S_{1d}S_{2a}}$, $U_{3}=\frac{S_{2a}}{S_{1a}S_{2d}-S_{1d}S_{2a}}$,

$U_{4}=-\frac{S_{1a}}{S_{1a}S_{2d}-S_{1d}S_{2a}}$;

$S_{1a}=-1.0000000000 A+0.8581959666 B-12.35961914 C$,

$S_{1b}=-1.0000000000D$, $S_{1c}=+2.005226046 E-12.35961914 G+10.60697530 H$,

$S_{1d}=-11.74232364 B$, $S_{1e}=0$,

$S_{1f}=-6.312477510 E-5700.676306 H$, $S_{1g}=3.994625840 F$, $S_{1h}=-67.36709586 F$,

$S_{1i}=201.8072370 F$, $S_{1j}=-381.7008418 F$,$S_{1k}=0.7829242108I$;

$S_{2a}=1.873752475 B$, $S_{2b}=0$, $S_{2c}=+0.5091766256 E+23.15886696 H$,

$S_{2d}=-1.000000000 A-13.29402727 B-485.4811154 C$, $S_{2e}=-1.000000000 D$,

$S_{2f}=+2.018651170 E-485.4811154 G-6453999206 H$, $S_{2g}=8.721714324 F$, $S_{2h}=-89.63073490 F$,$S_{2i}=257.8009342 F$, $S_{2j}=-432.1411636 F$, $S_{2k}=0.4339831449I$.
